# Development of DARPin T cell engagers for specific targeting of tumor-associated HLA/peptide complexes

**DOI:** 10.1016/j.isci.2025.113926

**Published:** 2025-11-03

**Authors:** Natalia Venetz-Arenas, Tim Schulte, Sandra Müller, Karin Wallden, Stefanie Fischer, Tom Resink, Nadir Kadri, Maria Paladino, Nicole Pina, Filip Radom, Denis Villemagne, Sandra Bruckmaier, Andreas Cornelius, Tanja Hospodarsch, Evren Alici, Hans-Gustaf Ljunggren, Benedict J. Chambers, Xiao Han, Renhua Sun, Marta Carroni, Victor Levitsky, Tatyana Sandalova, Marcel Walser, Adnane Achour

**Affiliations:** 1 Molecular Partners AG, Schlieren-Zurich, Switzerland; 2 Science for Life Laboratory, Department of Medicine Solna, Karolinska Institute & Division of Infectious Diseases, Karolinska University Hospital, Stockholm, Sweden; 3 Department of Biochemistry and Biophysics, National Bioinformatics Infrastructure Sweden, Science for Life Laboratory, Stockholm University, Box 1031, 17121 Solna, Sweden; 4 Science for Life Laboratory, CryoEM, Sweden, Department of Biochemistry & Biophysics, Stockholm University, Solna, Sweden; 5 Center for Hematology and Regenerative Medicine, Department of Medicine Huddinge, Karolinska Institutet, 17121 Huddinge, Sweden; 6 Center for Infectious Medicine, Department of Medicine Huddinge, Karolinska Institute, Karolinska University Hospital, Stockholm, Sweden

**Keywords:** Immunology, Structural biology, Cancer

## Abstract

The balance between affinity and specificity in T cell receptor (TCR)-dependent targeting of HLA-restricted tumor-associated antigens presents a significant challenge for immunotherapy development. T cell engagers that circumvent these limitations are therefore of particular interest. We established a process to generate bispecific designed ankyrin repeat proteins (DARPins) that simultaneously target HLA-I/peptide complexes and CD3e. These high-affinity T cell engagers elicited CD8^+^ T cell activation against tumor targets with strong peptide specificity, as confirmed by X-scanning mutagenesis and functional killing assays. A cryo-EM structure of the ternary DARPin/HLA-A∗0201/NY-ESO1_157-165_ complex revealed a rigid, concave DARPin surface spanning the full length of the peptide-binding cleft, contacting both α-helices and the peptide. The present findings reveal promising immuno-oncotherapeutic approaches and demonstrate the feasibility of rapidly developing DARPins with high affinity and specificity for HLA/peptide targets, which can be readily combined with a new generation of anti-CD3e-specific DARPins.

## Highlights

• High-affinity HLA-A∗0201/NY-ESO1157-165 DARPins isolated in four selection rounds
• Developed bispecific DARPins targeting HLA-I/peptide and CD3ε simultaneously
• T cell engagers activated CD8^+^ T cells with strong peptide specificity
• Cryo-EM showed DARPin spans peptide-binding cleft, contacting helices and peptide

## Graphical abstract

**Figure:**
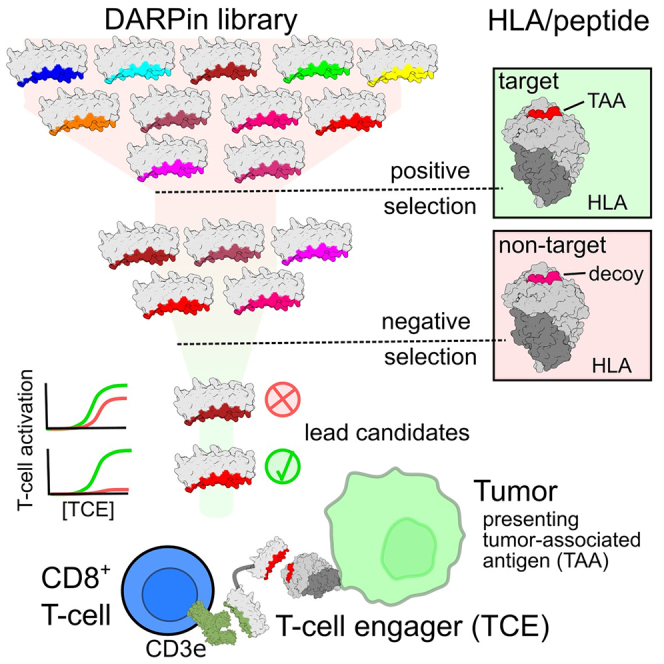

## Introduction

Technological advances in mass spectrometry and related methods have greatly enhanced our capacity to identify and map HLA-restricted tumor-associated antigens (TAAs) and neoantigen repertoires suitable as targets for CD8^+^ cytotoxic T lymphocyte (CTL)-mediated cancer immunotherapies. Moreover, the ability to direct adaptive immune responses through strategies, such as peptide vaccination^1^^,^^2^^,^^3^^,^^4^^,^^5^ or adoptive T cell transfer,^6^^,^^7^ has shown promising results in several malignancies.^8^^,^^9^^,^^10^^,^^11^ In particular, the design of TCR-like antibodies, modified TCRs, or various T cell engagers (TCEs) with enhanced affinities for tumor-specific HLA/peptide complexes allows for individualized therapeutic approaches.^7^^,^^12^^,^^13^ For example, engineered high-affinity, soluble TCRs specific to the melanoma-associated antigen gp100 or the more widely cancer-associated NY-ESO1 peptides have demonstrated success in preclinical models and clinical trials with respect to specificity and potency.^8^^,^^9^^,^^10^^,^^11^^,^^14^ Despite these clinical successes, many patients fail to develop adequate responses, acquire resistance, or both.^15^ Consequently, there is a clear need to explore additional novel approaches to further improve clinical outcomes.

The widespread development of peptide-based immunotherapies is partially hindered by immunological phenomena such as T cell tolerance and exhaustion. T cell tolerance must be considered because many TAAs are derived from endogenous proteins. The lack of therapeutic efficacy observed in certain studies could be attributed to tolerance induction in the available T cell repertoire toward HLA-I in complexes presenting dominant TAAs.^16^^,^^17^^,^^18^ In addition to the affinity-limiting effect of thymic negative selection on TCRs displayed by HLA/TAA-restricted T cells, these populations may be further functionally suppressed by the tumor microenvironment or become exhausted due to cancer immunoediting.^19^^,^^20^ Furthermore, TCRs are relatively weak binders that, compared to high affinity antigen-specific antibodies, recognize HLA alleles in complex with a limited pool of peptides.^12^^,^^21^ In practice, however, the weaker affinity of TCRs can be overcome through *in vitro* selection and affinity maturation strategies to obtain affinity-matured TCRs, TCR-like antibody binders,^7^^,^^22^ or altered peptide ligands.^23^^,^^24^^,^^25^^,^^26^^,^^27^ Although TCRs can display exceptional specificity for an HLA/peptide complex and exhibit high sensitivity to conservative mutations, they may also bind multiple other HLA/peptide complexes through cross-recognition, potentially leading to off-target effects.^28^^,^^29^ This nonspecific recognition can be exacerbated by gains in affinity,^30^^,^^31^ as illustrated by the high-affinity TCR developed against cancer-associated HLA-A∗0101/MAGE-A3 target. The clinical administration of T cells expressing this high affinity-enhanced TCR, which showed promising *in vitro* specificity, induced lethal off-target responses against an HLA-A∗0101-restricted titin-derived peptide on cardiomyocytes.^32^^,^^33^

We hypothesized that designed ankyrin repeat proteins (DARPins) could serve as alternative antigen recognition molecules to circumvent these limitations. The intrinsic rigidity of DARPins, combined with their longitudinal and transversal dimensions,^34^^,^^35^ suggested that these molecules could effectively engage the entire length of the peptide binding cleft of an HLA/peptide. The versatility of DARPins, along with their high affinity and specificity, has led to their successful use as therapeutics against a wide variety of targets.^36^^,^^37^^,^^38^ DARPins, which are based on an ankyrin repeat scaffold, create an elongated solenoid fold that creates a rigid, concave interface for target binding.^34^^,^^39^^,^^40^ In contrast to antibodies and TCRs, DARPins do not contain cysteine residues. As a result, they can both be readily expressed in soluble form in *Escherichia coli* at very high yields and exhibit favorable biophysical properties.^41^^,^^42^

In this study, we developed and characterized bispecific T cell engagers by combining two highly specific DARPin molecules to target the tumor-associated HLA-A∗0201/NY-ESO1_157-165_ complex and CD3ε. We demonstrated that DARPins can serve as a novel scaffold for TCEs that combine high specificity, potent T cell activation, and minimal off-target activity. The cryo-EM structure of the lead DARPin candidate in complex with HLA-A∗0201/NY-ESO1_157-165_ further revealed that the DARPin binding surface spans the entire length of the HLA peptide binding cleft, contacting both helices and peptide. The results provide compelling evidence that DARPin-based TCEs can overcome the key limitations of existing TCR- and antibody-based approaches for targeting intracellular tumor antigens presented by HLA molecules.

## Results

### Identification and validation of designed ankyrin repeat proteins with high affinity and specificity for HLA-A∗0201/NY-ESO1_157-165_

**Figure 1:**
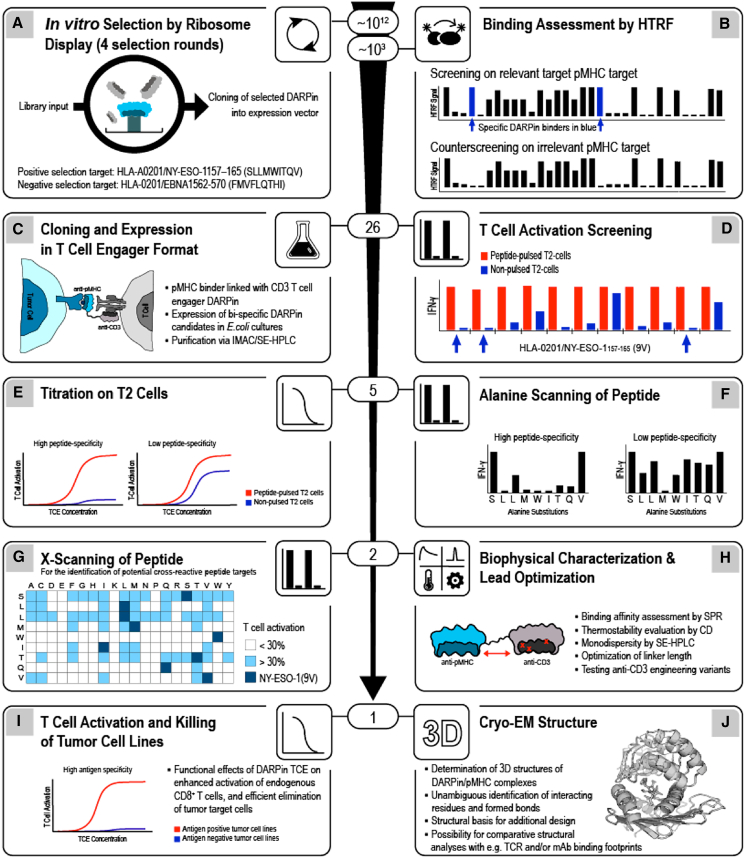
Generation of HLA/peptide-specific DARPins A general workflow for creating and selecting bispecific DARPin T cell engagers (TCEs) is shown, focusing on HLA-A∗0201/NY-ESO1_157-165_ as proof-of-concept. (A) A physical DARPin library (approximately 10^12^ complexes) was used for *in vitro* ribosome display selections of HLA-A∗0201/NY-ESO1_157-165_(9V)-specific DARPin binders. Negative selection was performed against HLA-A∗0201/EBNA1_562-570_. (B) Homogenous Time-Resolved FRET (HTRF) screening of approximately 1,000 selected DARPin identified binders to HLA-A∗0201/NY-ESO1_157-165_(9V), with HLA-A∗0201/EBNA1_562-570_ as a negative control. (C) A total of 26 HLA-A∗0201/NY-ESO1_157-165_(9V)-specific DARPin binders were chosen, and each was fused to a CD3ε-specific DARPin to generate DARPin TCEs. (D) T cell activation assays using peptide-pulsed or unpulsed T2 cells, combined with CD8 T cells and three concentrations of each DARPin TCE, yielded five top candidates (Figure S1). (E) The specificity and potency of DARPin TCEs were confirmed with a panel of HLA-A∗0201^+^ tumor cell lines expressing the wild-type epitope NY-ESO1_157-165_ (9C) (Figure 2). (F) Alanine scanning across all peptide residues demonstrated the TCE specificity for the entire epitope (Figure 2C). (G) X-scanning T cell activation assays further identified possible substitution residues at each peptide position. These data were used to query the Swiss-Prot database for potential cross-reactive human peptide-HLA complexes (Figure 3). (H) Biophysical characterization of the two lead DARPin TCE candidates included varying linker lengths and CD3ε-binding affinities (Figures 4 and S2). (I) The remaining lead was tested in T cell activation and cytotoxic assays against antigen-positive and antigen-negative tumor cell lines (Figure 5). (J) The three-dimensional structure of the lead DARPin candidate in complex with HLA-A∗0201/NY-ESO1_157–165_(9V) determined to ∼3 Å resolution by cryo-electron microscopy (Figure 6). See also Figures S1 and S2.

We initiated this study by selecting HLA-A∗0201/NY-ESO1_157–165_(9V) (SLLMWITQV) as a target, because the modification of the anchor residue p9C in HLA-A∗0201-restricted NY-ESO1_157165_ (SLLMWITQC) to valine markedly improves HLA/peptide complex stability and TCR recognition without altering the peptide conformation.^23^ Using four different DARPin ribosome display mRNA libraries, each with a physical diversity of approximately 10^12^ variants, we performed four rounds of selection and counter-selection against HLA-A∗0201/NY-ESO1_157-165_(9V) and the negative control HLA-A∗0201/EBNA1_562-570_ (FMVFLQTHI), respectively (Figure 1A). Candidate DARPins were screened using homogenous time-resolved FRET (HTRF) to detect binding to HLA-A∗0201/NY-ESO1_157–165_(9V) (Figure 1B). Off-target binding was assessed using the negative controls HLA-A∗0201/EBNA1_562-570_ and HLA-A∗0201/NY-ESO1_157-165_(9VAA) (SLLAAITQV), in which the major TCR-interacting peptide residues p4M and p5W^43^ were mutated to alanine. All DARPin binders that generated HTRF signals more than 50-fold higher for HLA-A∗0201/NY-ESO1_157-165_(9V) compared to both negative controls were linked to an anti-CD3ε DARPin^44^ to create bispecific TCE constructs (Figure 1C, Table S1).

### Development and validation of bispecific designed ankyrin repeat protein T cell engagers targeting HLA-A∗0201/NY-ESO1_157-165_ and CD3ε

The ability of 26 different DARPin TCEs to mediate highly specific and potent CD8^+^ T cell activation was assessed by measuring intracellular IFNγ levels in the presence of either NY-ESO1_157-165_(9V)-pulsed TAP-deficient T2 cells or unpulsed T2 cells (Figures 1D and S1). Subsequently, the capacity of five selected DARPin TCEs to elicit wild-type NY-ESO1_157-165_ (9C)-specific activation of CD8^+^ T cells was evaluated by co-incubating T cells and each TCE with HLA-A∗0201^+^ tumor cell lines expressing NY-ESO1_157-165_ (9C) (Figure 1E). These experiments demonstrated the unambiguous capacity of the five selected DARPin TCEs to recognize the wild-type epitope at physiological ligand levels.

**Figure 2:**
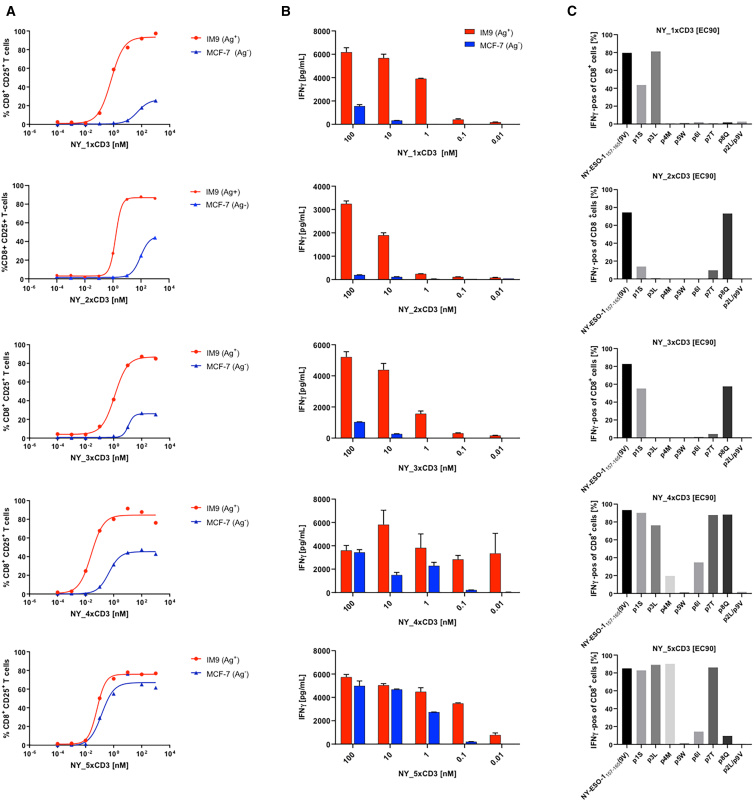
DARPin TCEs induce robust and highly specific CD8^+^ T cell responses against HLA-A∗0201/NY-ESO1_157-165_ Prior to linker and CD3 binder optimization, we made use of an intermediate 24 amino acid-long linker (standard linker length for most of the developed DARPin constructs), and a parental CD3 binder from where the stability-improved versions CD3-v1, CD3-v2, and CD3-v3 originated. This parental CD3 DARPin binder has an affinity in the range of the version 2 presented in this article, with a K_D_ value of approximately 14 nM (A and B) The HLA-A∗0201^+^/NY-ESO1^+^ (Ag^+^) IM9 and HLA-A∗0201^+^/NY-ESO1^-^ (Ag^−^) MCF-7 tumor cell lines were incubated with PBMCs for 48 h in the presence or absence of each DARPin TCE. T cell activation was evaluated by measuring CD25 expression on CD8^+^ T cells (A) and IFNγ release (B). (C) T2 cells were pulsed with 1 μM alanine-substituted peptide variants of the NY-ESO1_157–165_(9V) peptide and then incubated with effector CD8^+^ T cells plus each DARPin TCE, followed by the quantification of IFNγ positive CD8^+^ T cells. NY_1xCD3, NY_2xCD3, and NY_3xCD3 were highly sensitive to mutations spanning the entire peptide, whereas NY_4xCD3 and NY_5xCD3 were predominantly sensitive to changes at residues p4-p6. (A–C) Results are representative of at least three independent experiments. For panel B, error bars represent the standard deviation obtained in a single experiment. See also Table S1, Figures S3 and S4.

Notably, the five lead DARPin TCE candidates, henceforth referred to as NY_1xCD3 through NY_5xCD3, induced a significant, dose-dependent increase in the CD8^+^ T cell activation marker CD25 (Figure 2A) and in IFNγ production by CD8^+^ T cells (Figure 2B) when co-incubated with HLA-A∗0201^+^/NY-ESO1_157-165_ (9C)-positive tumor cells. In contrast, CD25 and IFNγ expression levels remained negligible in the presence of NY-ESO1-negative tumor target cells for most of these tested DARPin TCEs. We further evaluated each DARPin TCE’s specificity using T2 cells pulsed with single alanine variants of NY-ESO1_157-165_ (Figures 1F and 2C). The results showed that NY_1xCD3, NY_2xCD3, and NY_3xCD3 displayed high specificity across most peptide positions (Figure 2C). In contrast, NY_4xCD3 and NY_5xCD3 exhibited nonspecific cytokine production (Figures 2A and 2B) and were sensitive to mutations at residues p4-p6 and p5-p6, respectively (Figure 2C). Given their small but significant differences in peptide specificities (Figure 2C), we selected NY_1xCD3 and NY_2xCD3 for further studies. NY_3xCD3 was discarded because its peptide activation profile was largely redundant with that of NY_2xCD3 (Figure 2C).

HPLC-size exclusion chromatography analyses confirmed that both lead TCE candidates were monodisperse in solution, with no evidence of aggregation or oligomerization (Figure S2). In addition, both DARPin TCEs were highly thermostable, exhibiting melting temperatures of 70°C and 60°C for NY_1xCD3 and NY_2xCD3, respectively (Figure S2). Surface plasmon resonance measurements of NY_1xCD3 and NY_2xCD3 binding to HLA-A∗0201^+^/NY-ESO1_157-165_(9V) revealed different binding kinetics, despite comparable K_D_ values in the single-digit nanomolar range (Figure S2).

We further assessed the potency and specificity of these DARPin TCE candidates in cytotoxic assays using CD8^+^ T cells as effector cells against multiple target cell lines. Consistent with the T cell activation screening, both NY_1xCD3 and NY_2xCD3 provoked specific lysis of NY-ESO1_157-165_(9V)-pulsed T2 cells (Figure S3A and S4A). Moreover, the co-incubation of CD8^+^ T cells with NY-ESO1_157-165_ (9C)-positive killing of antigen-positive but not antigen-negative targets (Figures S3B and S4B). Finally, both tumor target cell lines in the presence of NY_1xCD3 or NY_2xCD3 resulted in efficient and specific NY_1xCD3 and NY_2xCD3 mediated robust cytotoxicity against NY-ESO1-transduced MCF-7 cells (Figures S3C and S4C).

### High specificity of designed ankyrin repeat proteins T cell engagers to NY-ESO1_157-165_ and absence of cross-reactivity

**Figure 3:**
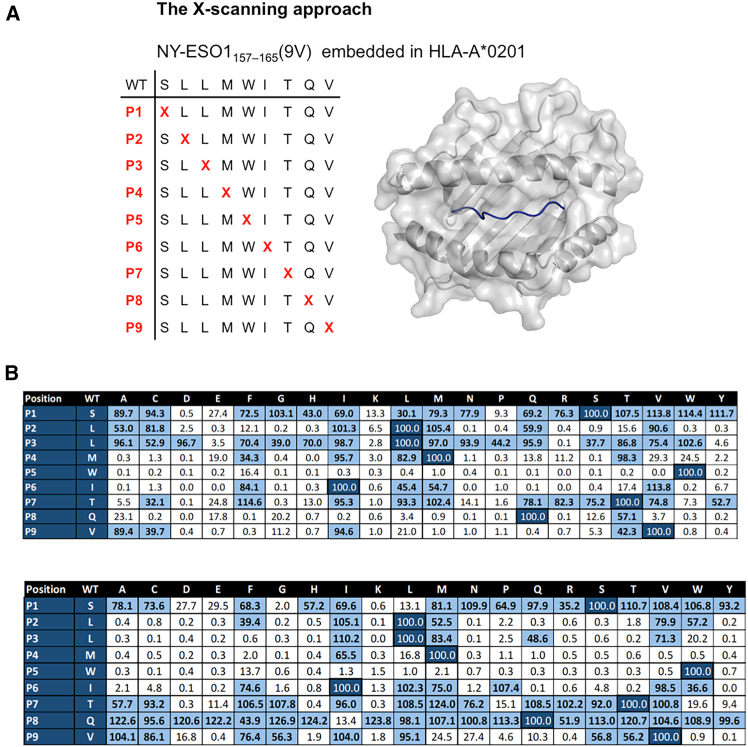
X-scanning mutagenesis reveals the high specificity of DARPin TCEs for HLA-A∗0201/NY-ESO1_157-165_ (A) An X-scanning mutagenesis approach was used to systematically replace each residue of the NY-ESO1_157–165 peptide with every possible amino acid variant, assessing the specificity of DARPins NY_1 and NY_3 for HLA-A∗*0201/NY-ESO1*_*157–165*_. *The peptide is shown as a blue ribbon within the peptide-binding groove of HLA-A∗*0201, whose surface is depicted in transparent gray. (B) T2 cells were pulsed with each mutated peptide and then incubated with effector CD8^+^ T cells in the presence of each DARPin TCE at its EC_90_ concentrations. The percentage of IFNγ positive CD8^+^ T cells is shown. Data from two independent experiments were averaged and normalized to 100% for the wild type residue at each position (dark blue). Values exceeding 30%, indicating substantial T cell activation, are highlighted (light blue). Each DARPin TCE was tested in two independent replicates. See also Table S2.

X-scanning mutagenesis, in which each peptide residue is substituted with all possible amino acids, revealed that NY_1xCD3 and NY_2xCD3 interact with multiple NY-ESO1_157-165_(9V) residues, and that few mutations are tolerated at positions p4-p8 and p2-p6 for NY_1xCD3 and NY_2xCD3, respectively (Figures 1G and 3). Mutations at peptide residues p4M and p5W, both recognized by TCR 1G4 in ternary complexes,^23^ in most cases, abolished recognition by the DARPin TCEs (Figure 3). Recognition by NY_1xCD3 is primarily governed by central and C-terminal peptide residues (p4-p9), whereas NY_2xCD3 recognition depends mainly on central and N-terminal residues (p1-p7). This finding aligns with previous alanine scanning results (Figure 2C). Crucially, HLA-A∗0201/NY-ESO1_157-165_-specific DARPins bind across the entire length of the epitope, with most substitutions significantly reducing their T cell activation capacity. As a result, the engagement of even closely related peptides would be limited or not tolerated.

To mitigate potential cross-reactivity, we used X-scanning mutagenesis binding profiles to identify, *in silico*, a limited set of potentially recognized peptide sequences (Table S2). All identified epitopes were tested for their ability to mediate CD8^+^ T cell activation when pulsed onto T2 cells in the presence of either NY_1xCD3 or NY_2xCD3. None of the epitopes induced cross-reactivity with NY_1xCD3, and only two cross-reactive epitopes were identified for NY_2xCD3. Specifically, SLLMWLTPL provoked significant CD8^+^ T cell recognition with NY_2xCD3, whereas TLLIWLFEV induced only weak cross-reactivity (Table S2).

### Engineered designed ankyrin repeat proteins T cell engagers with improved potency and maintained specificity

**Figure 4:**
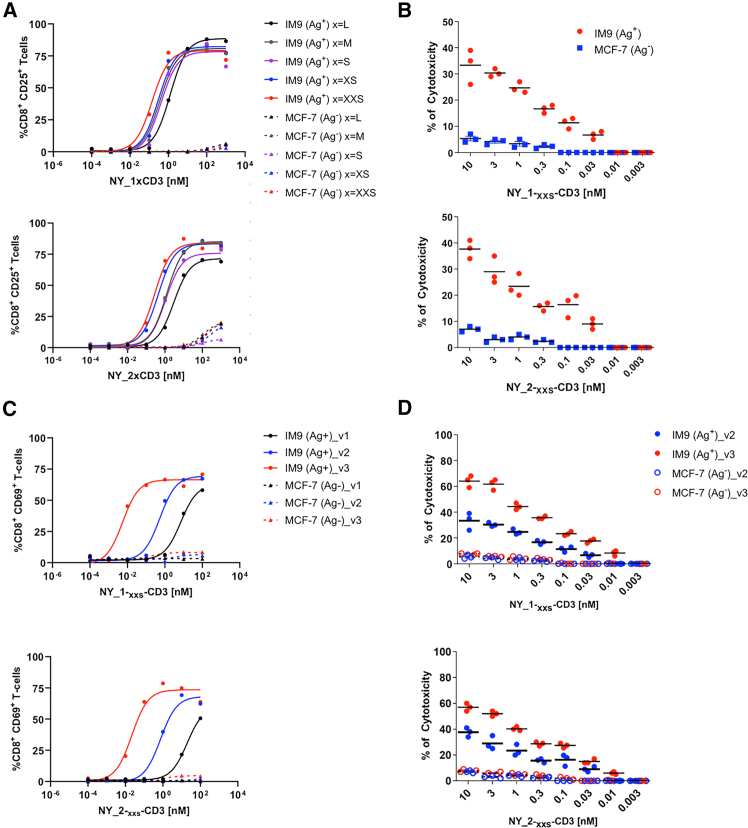
Engineering of linker length and CD3ε affinity in DARPin TCEs increases potency without affecting specificity HLA-A∗0201^+^/NY-ESO1^+^ (Ag^+^) IM9 or HLA-A∗0201^+^/NY-ESO1^-^ (Ag^−^) MCF-7 tumor cell lines were incubated with PBMCs for 48 h in the presence or absence of (A and B) NY_1xCD3 or NY_2xCD3 with different linkers (L to XXS), or (C and D) sequence optimized versions (v) of NY_1xCD3 or NY_2xCD3 (v1 to v3). The effects of each modification on potency were evaluated by measuring the T cell activation markers CD25 (A) and CD69 (C). IM9 and MCF-7 cells were incubated with effector CD8^+^ T cells in the presence or absence of (B) NY_1xCD3 or NY_2xCD3 carrying the optimal linker length sequence (XXS) or (D) the optimized variants (v2 and v3). The percentage of specific lysis of different tumor cell lines was determined using classical chromium release assays. (A–D) All results are representative of at least two independent experiments. For panels B and D, experiments were run in triplicate. See also Table S3.

We further engineered NY_1xCD3 and NY_2xCD3 to enhance their potency without compromising their specificity (Figure 1H). First, linkers of various lengths (Table S3) were inserted between the HLA-A∗0201/NY-ESO1_157-165_- and the CD3ε-specific DARPin domains (Figures 1H and 4A). These new constructs were then evaluated for their capacity to induce CD8^+^ T cell activation against either the HLA-A∗0201^+^/NY- ESO1_157-165_ (9C)-positive cell line IM9 or the HLA-A∗0201^+^/NY-ESO1_157-165_ (9C)-negative cell line MCF-7 (Figures 1I and 4A). DARPin TCE featuring the shortest (six-amino-acid) linkers triggered the most potent CD8^+^ T cell responses (Figures 4A and 4B, Table S3). Subsequently, we generated affinity-matured CD3ε-specific DARPins with affinity values ranging from 6 to 35 nM (Figure 4C, Tables S1, and S3). Combining the highest affinity CD3ε-specific binders paired with the shortest linker produced the most potent CD8^+^ T cell activation and cytotoxicity, without loss of specificity (Figures 4C and 4D, Table S3).

**Figure 5:**
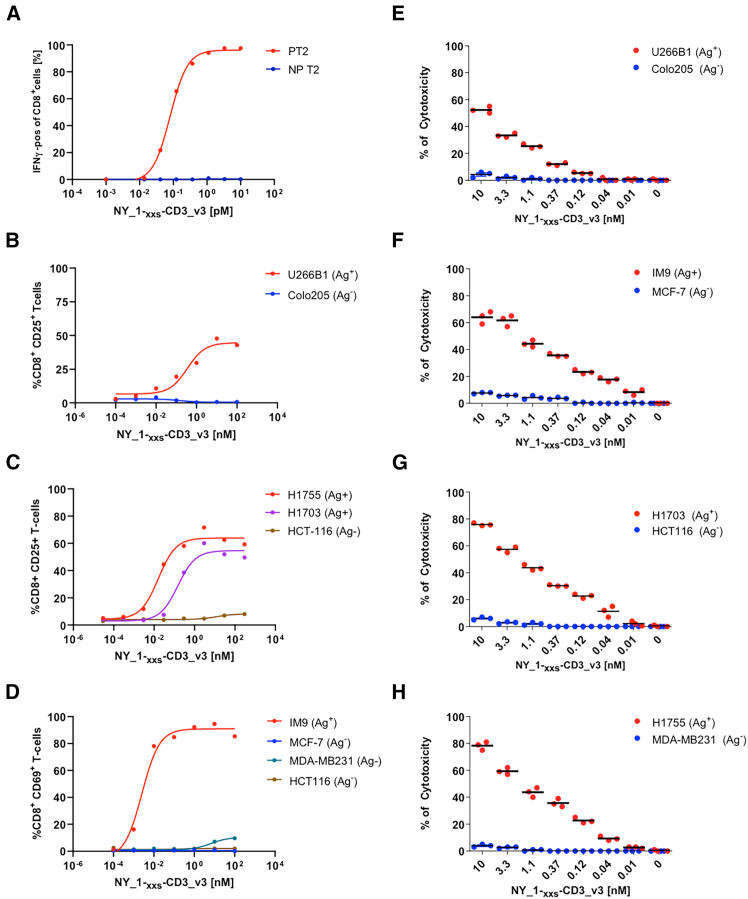
The engineered NY_1xCD3_v3 TCE induces potent and specific T cell-mediated killing of HLA-A∗0201^+^/NY-ESO1_157-165_ (9C)^+^ target cells (A) T2 cells pulsed with 1 μM NY-ESO1_157–165_(9V) (PT2) or unpulsed T2 cells (NP T2) were incubated with effector CD8^+^ T cells in the presence or absence of NY_1xCD3_v3. Intracellular IFNγ levels in CD8^+^ T cells are shown. (B–H) The HLA-A∗0201^+^/NY-ESO1^+^ (Ag^+^) U266-B1, IM9, NCI-H1755, NCI-H1703, and HLA-A∗0201^+^/NY-ESO1^-^ (Ag^−^) tumor cell lines Colo-205, MCF-7, MDA-MB231, and HCT-116 were incubated with PBMCs (B–D) or CD8^+^ T cells (E–H) in the presence or absence of NY_1xCD3_v3. Panels (B–D) display the levels of the T cell activation markers CD25 and CD69 in CD8^+^ T cells, while panels (E-H) present the percentages of specific lysis determined by chromium release assays for each tumor cell line. (A–H) All results are representative of at least two independent experiments. For panels E to H, experiments were run in triplicate.

We then evaluated the most potent lead TCE, NY_1xCD3_v3, with a broader panel of tumor cell lines expressing physiological levels of HLA-A∗0201^+^/NY-ESO1_157-165_ (9C) (Figures 1I and 5). The addition of NY_1xCD3_v3 induced a dose-dependent increase in the activation markers CD25 (Figures 5A and 5B) and CD69 (Figure 5C) on CD8^+^ T cells in the presence of all HLA-A∗0201/NY-ESO1_157-165_ (9C)-positive tumor cell lines, but not in antigen-negative cells. Cytotoxic assays on the same set of tumor cell lines further confirmed that NY_1xCD3_v3 mediated highly efficient, HLA-A∗0201/NY-ESO1_157-165_ (9C)-specific killing (Figures 5D–5F). In contrast, no significant lysis was observed for NY-ESO1_157-165_ (9C)-negative tumor cell lines, even at high DARPin concentrations (Figures 5D–5F).

### Designed ankyrin repeat proteins NY_1 spans the entire HLA/peptide binding cleft

**Figure 6:**
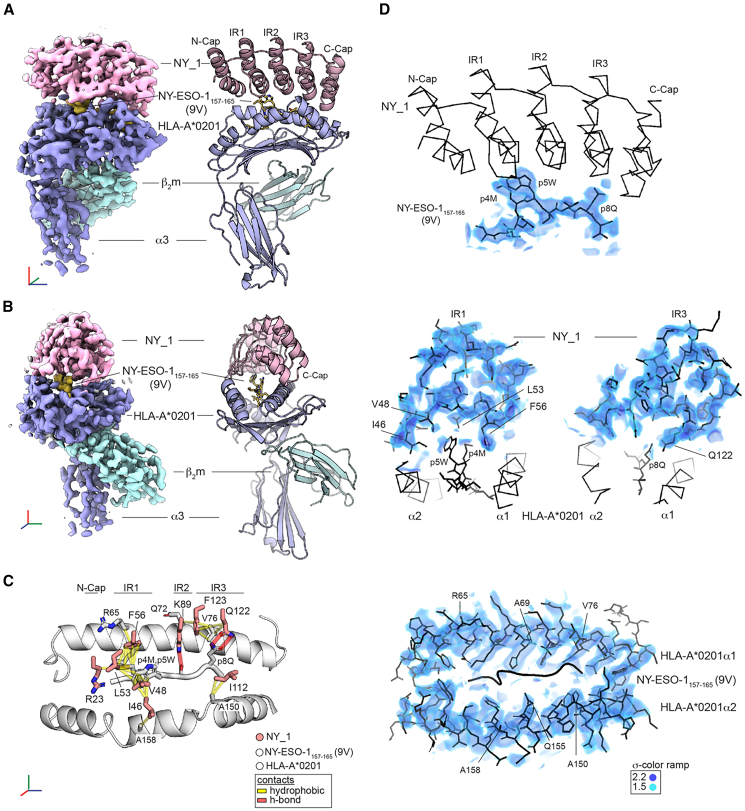
Cryo-EM structure of the NY_1/HLA-A∗0201/NY-ESO1_157-165_(9V) complex (A) Final map and model of the DARPin NY_1 (light pink) in complex with HLA-A∗0201/NY-ESO1_157-165_(9V). The HLA class I heavy chain, the β_2_ microgloulin (β_2_m), and the peptide are shown in light-blue, cyan, and yellow, respectively. The cap and internal repeat (IR) elements of the NY_1 DARPin are labeled. A coordinate frame with blue, green, and red axes indicates the orientation of the complex. (B) The same model and map as in (A), shown from a different orientation. (C) NY_1 makes a limited amount of specific close contacts with HLA-A∗0201/NY-ESO1_157-165_(9V). The peptide binding cleft of HLA-A∗0201 and the NY-ESO1_157-165_(9V) epitope are depicted in white. Residues that interact with NY_1 are rendered as sticks. DARPin residues contacting the HLA-peptide complex are shown in light pink. R23 (N-cap); I46, V48, L53 and F56 (IR1); K89 (IR2); I112, Q122 and F123 (IR3). Hydrophobic contacts and hydrogen bonds are indicated by yellow and red lines, respectively. (D) The final map is displayed for selected regions of the model, outlined in black. The map is presented for the peptide (top), IR1 and IR3 (middle), and MHC α1 and α2 helices (bottom). Visual guides without the map are provided as follows: NY_1 as ribbon (top), MHC α1 and α2 helices plus peptide as ribbon and stick (middle), and the peptide as a cartoon (bottom). Orientations match those in (A)–(C). The density is visualized as volume with the indicated color ramp. Additional maps, Fourier shell correlation curves, and map-model cross-correlation data are presented in Figures S5–S8. See also Tables S4 and S5, Figures S5, and S8.

**Figure 7:**
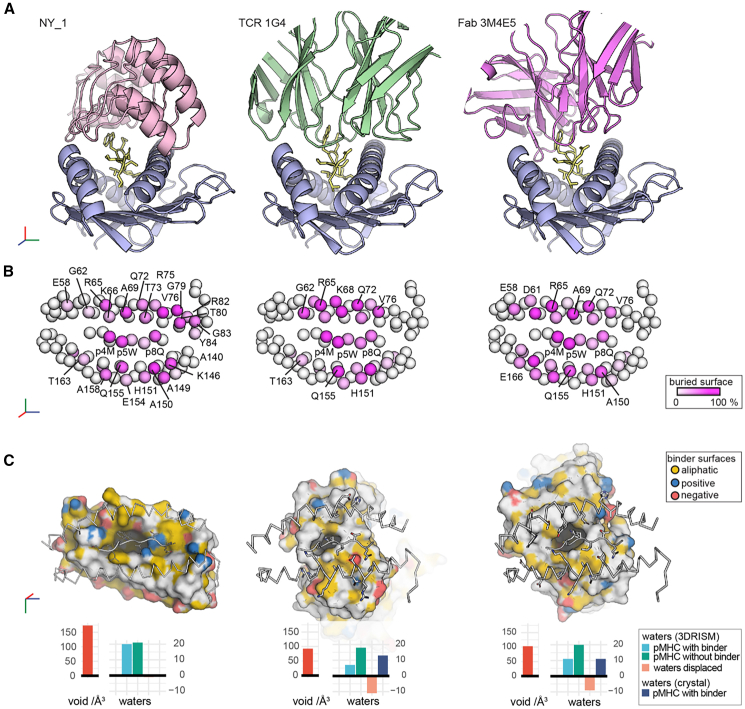
NY_1 spans the entire peptide binding cleft of HLA-A∗0201/NY-ESO1_157-165_(9V) The structure of NY_1/HLA-A∗0201/NY-ESO1_157-165_(9V), determined in this study, compared to previously determined crystal structures of HLA-A∗0201/NY-ESO1_157-165_(9V) in complex with TCR 1G4 and Fab fragment 3M4E5. (A) DARPin NY_1 binds along the entire peptide binding cleft, spanning the distance between the two helices. In contrast, the TCR 1G4 and the Fab fragment 3M4E5 interact with HLA-A∗0201/NY-ESO1_157-165_(9V) via flexible CDR loops that primarily contact the central region of the NY-ESO1_157-165_(9V) epitope and specific HLA-A∗0201 residues on both helices. The HLA-A∗0201 heavy chain is shown in blue, and the peptide in yellow. The DARPin, TCR, and Fab fragment are colored in light pink, green, and violet, respectively. (B) The C_α_ atoms of HLA-A∗0201/NY-ESO1_157-165_(9V) residues are depicted as spheres colored according to their buried surface areas upon binding (white to pink scale). (C) Surface views highlight the hydrophobic pockets formed by TCR 1G4 and Fab 3M4E5 around key peptide residues p4M and p5W. The top view is rotated by 180°, and the atoms of each binder are shown in yellow (hydrocarbon groups without polar substitutions), red (oxygens of negatively charged residues), and blue (nitrogens of positively charged residues). The larger void observed at the NY_1/HLA-A∗0201/NY-ESO1_157-165_(9V) interface is predicted to accommodate additional water molecules (Figures S9 andS10). See also Figures S9–S11.

To elucidate the molecular basis for NY_1’s affinity and specificity toward HLA-A∗0201/NY-ESO1_157-165_(9V), we determined the three-dimensional structure of this ternary complex using single-particle cryo-electron microscopy at around 3 Å resolution (Figures 1J, 6, and S5; Table S4). Each of the five post-processed maps (Figures S6–S8) permitted unambiguous placement of the crystal structures of NY_1 (Table S5) and HLA-A∗0201/NY-ESO1_157-165_(9V)^23^ (Figures 6A and 6B). Model-based density modification^45^ enhanced map interpretability, yielding a more contiguous density in the DARPin region (Figures 6D and S6–S8). While the DARPin/HLA-peptide interface is well defined, the HLA-A∗0201 α3-domain and the solvent-exposed residues of the NY_1 helices appear less resolved (Figures 6A, S6, and S8). The structures of NY_1 alone and in complex with HLA-A∗0201/NY-ESO1_157-165_(9V) were highly similar, with an overall root-mean-square deviation of 0.4 Å. NY_1 binds on top of HLA-A∗0201 via its concave, elongated interaction surface, which runs along the entire length of the peptide-binding cleft. Each constituent helix in the cap elements and internal repeats (IRs) is oriented perpendicular to the HLA α1 and α2 helices (Figures 6A, 6B, and 7A). The N- and C-cap elements are positioned above the peptide’s N- and C-termini, respectively, while the IRs of NY_1 engage the central peptide region (p4M to p8Q) (Figures 6A and 6D). Notably, the spacing between the two HLA α−helices closely accommodates the entire width of NY_1 (Figure 6A). NY_1 interacts with residues on both the HLA heavy chain α-helices and the NY-ESO1_157-165_(9V) peptide (Figure 6C). The centrally positioned peptide residues p4M and p5W are surrounded by the hydrophobic NY_1 IR1-residues I46, V48, L53, F56, and the aliphatic portion of the N-cap residue R23 (Figures 6C and 7B). Additional hydrogen bonds formed between NY_1 residues IR2-K89 and IR3-Q122 and peptide residues p6I and p8Q, respectively (Figure 6C).

We compared the NY_1/HLA-A∗0201/NY-ESO1_157-165_(9V) structure with the ternary complexes of TCR 1G4 or the Fab 3M4E5 bound to the same HLA/peptide target^23^^,^^46^(Figure 7). Previous TCR structures complexed with HLA-A∗0201/NY-ESO1_157-165_, except for NYE_S3, generally show hot-spot contacts at p4M and p5W.^23^^,^^46^^,^^47^^,^^48^^,^^49^ In contrast, NYE_S3 binds an altered peptide conformation of NY-ESO1_157-165_(9V).^47^ A comparative distance-ramp analysis indicated that the CDR loops of 1G4 and 3M4E5 form tight interfaces with HLA-A∗0201/NY-ESO1_157-165_(9V) (Figure S9A). Despite its distinct scaffold, NY_1 occupies a molecular footprint similar in shape and position to that of 1G4, including contacts with the TCR-restriction triad formed by MHC residues R65, A69, and Q155^50^^,^^51^ (Figures 7A, 7B, and S9). Strikingly, contacts with the DARPin backbone are almost absent, whereas the backbone atoms of TCRs and Fab fragments contribute around 30–40% of the total binding interfaces (Figure S9A). NY_1 buries an interface area of around 1170 Å^2^, about 10% less than the ∼1300 Å^2^ area buried by 1G4 or 3M4E5 (Figure S9). All three binders contact both the peptide and the HLA α1 and α2 helices, albeit to differing extents (Figures 7B and S9). NY_1 and 3M4E5 feature larger footprints than the diagonal binding mode of 1G4, forming extended interfaces along the α1 and α2 helices (Figures 7B and S9).

NY_1/HLA-A∗0201/NY-ESO1_157-165_(9V) adopts a slightly less compact binding interface, generating a solvent-accessible void volume of around 175 Å^3^ along the peptide (Figures 7C and S10). By contrast, 1G4 and 3M4E5 form tight hydrophobic pockets around p4M and p5W, restricting the void volumes to around 100 Å^3^. Although our ∼3 Å cryo-EM data did not resolve water molecules, a solvation prediction algorithm^52^^,^^53^ suggests that the interface between NY_1 and HLA-A∗0201/NY-ESO1_157-165_(9V) is likely to contain more water molecules than the tighter interfaces formed of 1G4 and 3M4E5 (Figures 7C, S10, and S11). Such coordinated water molecules often play crucial roles in fine-tuning specificity and avidity in protein/ligand interactions,^54^^,^^55^^,^^56^ including the TCR recognition of HLA/peptide complexes.^57^^,^^58^^,^^59^ Furthermore, water-mediated “cushions” can lower the entropic costs of ligand binding, thereby significantly increasing affinity.^60^

## Discussion

Developing biological tools, such as soluble TCRs, TCR-like antibodies, and cellular therapeutics that target low-abundance tumor-associated HLA/peptide complexes can be challenging. Common issues include the relatively low affinity of these agents, potential cross-reactivity to similar or unrelated epitopes, and the complexity of manufacture and clinical administration. DARPin-based therapeutics offer the potential to overcome these limitations owing to their excellent biophysical properties and advantageous structural characteristics.^36^^,^^37^^,^^38^ At the outset of this study, we hypothesized that the compact and rigid binding domains of DARPins could be particularly well-suited for the size and relatively flat surface of an HLA/peptide complex. Although the flexible CDRs of TCRs or antibodies can sometimes lead to cross-reactivity and non-specific binding, the rigid scaffold and binding surface of DARPins typically mediate highly specific interactions with target proteins. The present study demonstrates that the DARPin molecule NY_1 binds along the entire length of HLA-A∗0201/NY-ESO1_157-165_, spanning the distance between the two α−helices flanking the peptide binding cleft. Although NY_1 engages structural features also bound by TCRs and TCR-like antibodies, its elongated scaffold allows additional contacts with residues at both termini of the HLA-A∗0201 peptide-binding cleft. Notably, this enlarged footprint may restrict cross-reactivity with other HLA/peptide complexes, as shown by X-scanning mutagenesis. Based on these findings, NY_1 may display increased specificity compared with TCR 1G4 and Fab 3M4E5, due to the absence of mobile and ligand-adaptable CDR loops, reliance on a few but specific sidechain-mediated contacts, and a larger water “cushion” at the interface.

It is well established that multiple mechanisms underlie the immunoregulatory function of cancer cells.^61^ Various strategies adopted during tumor progression promote immune escape, including the induction of checkpoint molecules or downregulation of MHC class I processing pathways. Tumor cells further exploit multiple immune cell types, such as regulatory T cells, neutrophils, and macrophages, to create an immunosuppressive tumor microenvironment. These properties, in combination with extracellular matrix formation and angiogenesis, facilitate immune escape, tumor progression, and resistance to therapy.^62^^,^^63^^,^^64^ Our approach in this study was to combine DARPins with high affinity and specificity toward CD3ε and HLA/TAA complexes, thus creating TCEs that can recruit, activate, and redirect CD8^+^ T cells to efficiently eliminate tumor cells. The functional data show that these TCEs mediate robust killing of a broad panel of HLA-A∗0201/NY-ESO1_157-165_ (9C)-positive tumor cells. By contrast, no killing was observed on NY-ESO1_157-165_ (9C)-negative tumor cell lines. However, the transfection of the HLA-A∗0201^+^/NY-ESO1 negative cell line MCF-7 with a construct encoding the full-length NY-ESO1 protein resulted in highly efficient killing by endogenous CD8 T cells in the presence of our TCEs. Additionally, the linker properties between the two DARPins domains and the affinity of the CD3ε-specific DARPin both influenced TCE specificity. We hypothesize that a shorter linker between the HLA/peptide- and CD3ε-binding DARPins may constrain the recruited CD8^+^ T cells to a closer proximity of the tumor cells, potentially enhancing immunological synapse formation. Another non-excluding explanation is that this configuration prolongs the contact half-life between CD8 T cells and tumor targets, allowing more time for effector function. Finally, we observed that the higher affinity of the final selection of CD3ε-specific DARPins could elicit significantly stronger synapse formation, thereby increasing CD8^+^ T cell activation. Crucially, this enhanced activation did not lead to nonspecific killing of NY-ESO1-negative tumor cells.

While this work represents an *in vitro* proof-of-concept, several aspects suggest encouraging *in vivo* potential for DARPin-based TCEs. Their small size and high stability may support good tumor penetration and ease of production, although the former could also result in rapid renal clearance, necessitating half-life extension strategies. Importantly, the observed high specificity to the HLA/peptide complex may help mitigate systemic off-target activation. Future *in vivo* studies will therefore be essential to evaluate pharmacokinetics, biodistribution, and safety, and to determine whether the strong specificity and functional killing demonstrated in this study can be translated into therapeutic potential in animal models. However, TCR- and antibody-based candidates employing a similar mechanism, such as bi-specific TCR-*anti*-CD3 constructs targeting NY-ESO-1/LAGE-1, have already proven their *in vivo* efficacy and tumor growth control using engineered TCRs directed against the same peptide.^65^ Furthermore, encouraging clinical outcomes have also been reported with tebentafusp, the first approved TCR therapeutic, which targets the gp100 peptide and redirects T cells via CD3 engagement for the treatment of uveal and malignant melanoma.^66^ It should also be noted that the first DARPin TCE clinical candidate, MP0533, which employs the same CD3 binding domain (CD3v2) used in our study, has demonstrated promising proof-of-concept data in an ongoing Phase 1/2a trial in patients with relapsed/refractory AML and MDS/AML.^44^

A common strategy for TCR-based therapies is to identify HLA/TAA-specific TCRs and subsequently increase their affinity through directed mutagenesis.^67^^,^^68^ Although such approaches have been efficacious in both preclinical and clinical studies, higher TCR affinity often correlates with increased cross-reactivity against unwanted targets.^69^^,^^70^ Furthermore, a previous study revealed that a chimeric antigen receptor derived from the TCR-like antibody 3M4E5 caused moderate lysis of HLA-A∗0201-expressing targets through enhanced T cell avidity, independent of the presented antigen and despite the high affinity of Fab 3M4E5 to HLA-A∗0201/NY-ESO1_157-165_. It was therefore necessary to reduce the affinity of this TCR-like antibody to more conventional TCR levels in order to preserve specificity.^71^ In contrast, the high-affinity DARPin TCEs developed here, selected from naive libraries in four rounds of *in vitro* selection, maintained very high specificity.

We tested in this study DARPin TCEs with varying linker lengths using a parental CD3 binder (CD3v2, intermediate affinity) (Table S3). The shortest linker enhanced potency, both T cell activation and T cell-mediated cytotoxicity, against NY-ESO-1-positive tumor cells, without increasing nonspecific activation against NY-ESO-1-negative cells. Following linker optimization, we evaluated CD3 variants with lower, similar, or higher affinity than the parental binder. The highest-affinity variant (CD3v3) further increased potency without elevating nonspecific activation. These *in vitro* results enabled the identification of the optimal combination of linker length and CD3 affinity, achieving robust T cell activation even against tumor cells expressing very low levels of pMHC targets.

Although the efficacy and safety of TCEs ultimately depend on both CD3 affinity and target-antigen density, recent studies have demonstrated that the attenuation of CD3 affinity can reduce cytokine release without markedly compromising tumor killing.^72^^,^^73^ Our findings suggest that combining high peptide/HLA specificity with an anti-CD3 DARPin could benefit from similar tuning, where modest CD3 engagement may improve safety without losing potency against low-antigen density targets. Structural data showing extensive peptide–HLA contacts indicate a strong avidity margin that may permit such attenuation. Comparable conclusions have been reported for bispecific antibodies where high target-arm affinity was essential for efficacy, while lowering CD3 affinity limited toxicity.^74^^,^^75^ More recent work with the multi-specific DARPin MP0533 further supports that avidity and antigen selectivity can be exploited to balance efficacy and safety.^44^

In conclusion, DARPins provide a promising platform for creating HLA/peptide-specific therapeutics with enhanced potency. Our workflow enables the rapid identification and isolation of lead DARPin candidates against both HLA class I and class II molecules in complex with TAAs or pathogen-derived epitopes. As illustrated by our bispecific DARPin TCEs, tumor or virus-specific DARPins can readily be combined with any of the CD3ε-specific DARPins to generate potent, targeted immune responses.

### Limitations of the study

While our results demonstrate the feasibility of rapidly generating high-affinity, HLA/peptide-specific DARPins and their potential for T cell engagement, several limitations should be noted. First, the study focused only on a single HLA allele (HLA-A∗0201) and one tumor-associated peptide, which may restrict its broader applicability across diverse patient populations. Second, although *in vitro* assays confirmed specificity and functional killing, the safety and efficacy of these constructs remain to be validated *in vivo*. Third, the structural and functional analyses were performed under controlled experimental conditions, which do not fully reproduce the complexity of the tumor microenvironment. As such, future studies will need to expand the range of HLA/peptide targets, assess off-target risks, and evaluate therapeutic potential in preclinical models.

## Resource availability

### Lead contact

Further information and requests for resources and reagents should be directed to and will be fulfilled by the lead contact, Prof. Adnane Achour (adnane.achour@ki.se).

### Materials availability

All unique materials and reagents generated in this study are available from the lead contact upon reasonable request.

### Data and code availability

• Data: The crystal structure of NY_1 was deposited under PDB: 9EPA. The cryoEM map has been deposited under EMDB: EMD-50336. The cryo-EM structural model of the ternary DARPin NY_1/HLA-A0201/NY-ESO1 complex has been deposited under PDB: 9FE1.
• Code: We have not produced any code crucial to the results of the study.
• All other items: All other data supporting the findings of this study are available from the corresponding author upon reasonable request.

## Acknowledgments

Parts of the computations were performed on the NSC Tetralith system, provided by the National Academic Infrastructure for Supercomputing in Sweden (NAISS), and at PReSTO, both funded by the 10.13039/501100004359Swedish Research Council under grant agreements no. 2022–06725 (NAISS) and no. 2018-06479 (PReSTO). Parts of this work were facilitated by the Protein Science Facility at Karolinska Institutet (http://ki.se/psf), and we thank Dr M. Moche for assistance. We also thank 10.13039/100011889Diamond Light Source for beamtime and the staff of beamline I24 for assistance and support with crystal testing and data collection. Funding A. Achour received financial support from Vetenskapsrådet (2021-05061), Cancerfonden (24 3775 Pj), Radiumhemmets Forskningsfonder (244092), and Insamlingsstiftelsen Cancer-och Allergifonden (244092) for this project. This work has been financially supported in part by Molecular Partners AG, which also provided some of the materials used in this research. A. Achour and T. Resink have been supported in part by the Vinnova NextGenNK Competence Center grant #2024-03709. Cryo-EM data were collected at the Cryo-EM Swedish National Facility, funded by the Knut and Alice Wallenberg, Family Erling Persson, and Kempe Foundations, SciLifeLab, Stockholm University, and Umeå University.

## Author contributions

A.A., V.L., and T.S. conceptualized the initial theoretical approach. A.A., V.L., T.S., N.V., M.W., T.Sch., K.W., S.M., and M.C. designed research and analyzed data. N.V., T.Sch., S.M., K.W., S.F., N.K., T.R., M.P., N.P., F.R., D.V., S.B., A.C., T.H., X.H., R.S., E.A., H.G.L., B.J.C., M.C., T.S., and M.W. generated and/or interpreted research results. AA wrote the first draft of the article, and N.V., T.S., T.R., S.M., K.W., M.C., T.Sch., B.J.C., E.A., and H.G.L. contributed to the finalization of the article.

## Declaration of interests

N.V., S.M., S.F., M.P., N.P., D.V., S.B., A.C., T.H., and M.W. are employed by Molecular Partners AG and hold options or shares in the company. V.L. was employed by Molecular Partners AG at the time of this research and holds options/shares in the company. All the other authors declare that they have no conflict of interest.

## STAR★Methods

### Key resources table

**Table:** 

REAGENT or RESOURCE	SOURCE	IDENTIFIER
**Antibodies**		

Live Dead stain aqua (for FACS)	Thermo	L34957
Live/Dead stain FITC (for FACS)	Thermo	L23101
APC mouse anti-human IFN-g (for FACS)	BD	554702; RRID:AB_398580
Pacific Blue mouse anti-human CD8 (for FACS)	BD	558207; RRID:AB_397058
CD8 Alexa 488 (for FACS)	BD	557696; RRID:AB_396805
CD25 PerCP Cy5.5 (for FACS)	ebio	45-0259-42; RRID:AB_11043556
CD69 Pe-Cy7	BD	560712; RRID:AB_1727509
CD4 efluor 450	ebio	48-0048-42; RRID:AB_2016674
CD3 PE	ebio	12-0037-42; RRID:AB_1272078
Penta-His AF488	Qiagen	35310; RRID:AB_3083465
anti-6His-D2 (HTRF assay)	Cisbio	61HISDLB; RRID:AB_2884027
1:400 anti-strep-Tb (HTRF assay)	Cisbio	610SATLB
RGS-His antibody (SPR)	Qiagen	34650; RRID:AB_2687898

**Bacterial and virus strains**		

E. coli BL21 (DE3)	Novagen	69450–3
E. coli XL1-Blue	Stratagene	200228

**Chemicals, peptides, and recombinant proteins**		

Peptide NY-ESO1_157-165_ (9V): SLLMWITQV	Genscript	Custom synthesis
Peptide NY-ESO1_157-165_(9VAA): SLLAAITQV	Genscript	Custom synthesis
Peptide EBNA-1_371_ FMVFLQTHI	Genscript	Custom synthesis
Peptides identified as potentially cross-reactive (listed in Table S2)	Genscript	Custom synthesis
Bacterial lysis buffer B-PERII	Thermo	78260
CD8^+^ T cell Isolation Kit, human	Milteny Biotec	130-096-495
human IL-2	Immunotools	11340025
Morpheus screen (protein crystallization)	Molecular Dimensions	MD1-46
BS(PEG)5 (cross-linking)	Thermo Fisher	A35396

**Deposited data**		

Cryo-EM map of the ternary DARPin NY_1/HLA-A0201/NY-ESO1 complex.	https://www.ebi.ac.uk/emdb/EMD-50336	EMD-50336
Cryo-EM structural model of the ternary DARPin NY_1/HLA-A0201/NY-ESO1 complex.	https://www.rcsb.org/structure/9FE1	9FE1
Crystal structure of DARPin NY_1	https://www.rcsb.org/structure/9EPA	9EPA

**Experimental models: Cell lines**		

T2 (Tumor cell line)	ATCC	CRL-1992
MCF-7 (Tumor cell line)	ATCC	HTB-22
U266B1 (Tumor cell line)	ATCC	TIB-196
IM9 (Tumor cell line)	ATCC	CCL-159
Colo-205 (Tumor cell line)	ECACC	87061208
HCT116 (Tumor cell line)	ATCC	CCL-247
NCI-H1755 (Tumor cell line)	ATCC	CRL-5892
NCI-H1703 (Tumor cell line)	ATCC	CRL-5889
MDA-MB231 (Tumor cell line)	ATCC	HTB-26
T cells isolated from PBMCs isolated from blood from healthy donors	N/A	

**Recombinant DNA**		

Codon-optimized HLA-A∗0201(HLA-A∗0201avi) construct comprising a linker (GSGGSGGSAGG) and the avi-biotinylation tag (GLNDIFEAQKIEWHE)	Eurofins Genomcs	Custom synthesis
pQE-30 expression vector	Qiagen	32915

**Software and algorithms**		

ScanProSite to identify cross-reactive peptides	Expasy prosite	https://prosite.expasy.org/scanprosite/
cryoSPARC (cryo-EM)	https://cryosparc.com/	3.3.1
Phenix (Model refinement)	https://www.phenix-online.org/	1.19
Coot (Model building)	https://www2.mrc-lmb.cam.ac.uk/personal/pemsley/coot/	0.9.5
UCSF ChimeraX (Model building and visualization)	https://www.rbvi.ucsf.edu/chimerax/	1.1.1
Isolde (Model refinement)	https://tristanic.github.io/isolde/	1.1.0
Molprobity (Model validation)	https://molprobity.biochem.duke.edu/	
PyMOL	Schrödinger	2.5.0
Arpeggio webserver	https://biosig.lab.uq.edu.au/arpeggioweb/	
PISA webserver	https://www.ebi.ac.uk/pdbe/pisa/	
CastP webserver	http://sts.bioe.uic.edu/castp/index.html?2pk9	
Ambertools	http://ambermd.org/GetAmber.php#ambertools	AmberTools21
Thermo Fisher Scientific EPU	https://www.thermofisher.com/se/en/home/electron-microscopy/products/software-em-3d-vis/epu-software.html	2.8.1
Rstudio	https://posit.co/download/rstudio-desktop/	1.3.1093

**Other**		

Size exclusion chromatography Superdex 200 HiLoad 16/600	Cytiva	28989335
Analytical size exclusion chromatography column Superdex 200 Increase 10/300 GL	Cytvia	28990944
Strep-Tactin Superflow high capacity columns (1 mL)	IBA lifescience	2-1252-001
IMAC HisPur Cobalt Spin Plates	Pierce/Thermo Fisher	90095
ZebaTm Spin Desalting Plates	Pierce/Thermo Fisher	89807
ProteOn GLC chip	Biorad	176–5011
96-well sitting-drop iQ plates (for crystallization)	sptlabtech	4150–05810
UltrAuFoil holey grids (R 0.6/1 gold foil on gold 300 mesh)	Quantifoil	

### Experimental model and study participant details

#### Human healthy volunteer-derived CD8^+^ T cells

Blood samples from anonymous healthy volunteers were obtained from the Karolinska University Hospital. Peripheral blood leukocytes were isolated by Ficoll density gradient centrifugation. CD8^+^ T cells were isolated using a human CD8^+^ T cell isolation kit (Milteny Biotec, Germany). Purity was confirmed by flow cytometry, and cells were seeded in RPMI 1640 with 10% FBS, and 1 μg/mL of phytohaemagglutinin (Thermofisher) at 10^6^ cells/ml. Cells were incubated at 37°C for three days, collected, washed and supplemented with 20 ng/mL of human IL-2 (Immunotools) was added. Cells were maintained for 4–6 days at a density of 10^6^ cells/ml in a 6-well plate.

#### Cell lines

IM-9, NCI-H1755, NCI-H1703, and U266 were used as HLA-A∗0201^+^/NY-ESO1^+^ cells lines. MDA-MB231, HCT116, Colo-205 and MCF-7 were used as HLA-A∗0201^+^/NY-ESO1^-^ cells lines. All cells were purchased from the American Type Culture Collection. MCF-7 transduced with NY-ESO1 were generated in house. HCT116 cells were maintained in McCOY’s 5a (Gibco), and T2, U266, Colo205 and IM-9 were maintained in RPMI 10 medium (Gibco). The remaining cell lines were maintained in DMEM (Lonza) medium. All media were supplemented with 10% heat-inactivated FCS (fetal calf serum, Biochrom), 2 mM L-glutamine, 1 mM HEPES and 100 U/ml penicillin/streptomycin. All cell culture flasks and plates were purchased from BD Falcon (Franklin Lakes, NJ). To pulse T2 cells, 10 μM of the NY-ESO1_157-165_(9V) peptide was added in RPMI medium (serum free) for 16 h at 37°C. Unpulsed cells were used as control. All cell lines (Table S7) were tested negative for mycoplasma.

#### Bacterial strains for cloning and protein expression

*E. coli* strains BL21 (DE3) and XL1-Blue were maintained following standard protocols, i.e., stored as 25% (V/V) glycerol stocks at −80°C and grown in TB or LB medium at temperatures ranging between 25°C and 37 °C. Single colonies were obtained by streaking bacterial cultures on TB or LB (1.5% w/V) agar plates. Selection antibiotics were used at concentrations recommended for the plasmids.

### Method details

#### Production of biotinylated MHC/peptide complexes for the selection of DARPin candidates

Complexes of HLA-A∗0201 with human β_2_-microglobulin (hβ_2_m) and each of the peptides NY-ESO1_157-165_(9V) (SLLMWITQV), NY-ESO1_157-165_(9VAA) (SLLAAITQV) and EBNA-1 (FMVFLQTHI), were produced as previously described.^76^^,^^77^ A codon-optimized HLA-A∗0201 (HLA-A∗0201avi) construct that comprised a linker (GSGGSGGSAGG) and an Avi-biotinylation tag (GLNDIFEAQKIEWHE) was expressed in *E. coli* BL21 (DE3) at 37°C as inclusion bodies (IB). The IB protein contents were purified and dissolved in 50 mM MES, 5 mM EDTA, 5 mM DTT, 8 M urea pH 6.5. HLA-A∗0201avi (or HLA-A∗0101avi) and hβ_2_m were refolded with each peptide at final concentrations of 25, 30 and 15 mg, respectively, per 500 mL in 50 mM Tris, pH 8.3, 230 mM L-arginine, 3 mM EDTA, 255 μM GSSG, 2.5 mM GSH, and 250 μM PMSF. For biotinylation, each refolded complex was concentrated to 7.5 mL, and the buffer was exchanged to 100 mM Tris pH 7.5, 150 mM NaCl, 5 mM MgCl2 pH 7.5 using PD10 columns. Avi-tagged heavy chains were then biotinylated by adding 5 mM ATP, 400 μM Biotin, 200 μM PMSF and 20 μg BirA enzyme. Each refolded, biotinylated complex was purified using size exclusion chromatography (Superdex 200 HiLoad 16/600, Cytiva) in PBS supplemented with 150 mM NaCl, 1 mM EDTA, 10% glycerol. Samples were concentrated to 1 mg/mL and flash-frozen in aliquots using liquid nitrogen.

#### Ribosome display selection of HLA-A∗0201/NY-ESO1_157-165_(9V)-specific DARPin candidates

Four DARPin libraries (N2C and N3C) were used in ribosome display selections^35^^,^^78^^,^^79^ against the refolded, biotinylated HLA/peptide complexes. Four selection rounds were performed for each pool. To direct DARPin binding toward the peptide rather than the HLA heavy chain scaffold, deselection steps were carried out using HLA-A∗0201/NY-ESO1_157-165_-9VAA and HLA-A∗0201/EBNA-1. Selection stringency was gradually increased to enrich high affinity binders. Nunc MaxiSorp 96-well microplates (Thermo Fisher Scientific, Zurich, Switzerland) were coated with 100 μL solution of 66 nM neutravidin in PBS and incubated at 4°C overnight. The next day, plates were washed three times with 300 μL PBST per well and blocked with 300 μL PBST-BSA for 1 h at 4°C, prior to deselection. After discarding the blocking solution, 100 μL of 50 nM biotinylated HLA/peptide deselection target in PBST-BSA (PBS pH 7.4, 0.05% Tween 20, 0.2% (w/v) BSA) was added to each well and plates were rotated at 700 rpm at 4°C for 1 h. During this incubation, *in vitro* mRNA translations were performed. Shortly after translations and generation of ternary ribosomal display complexes, solutions were discarded, and wells were washed three times with 300 μL PBST. Wells were then incubated with a washing buffer with tween (WBT) containing 0.2% (w/v) BSA: 50 mM Tris-HOAc (pH 7.5 at 4°C), 150 mM NaCl, 50 mM Mg(OAc)_2_, 0.05% Tween 20. For each deselection step, the washing buffer was discarded, and aliquots (150 μL for first selection round and 100 μL for rounds 2–4) of the translated ternary complexes were transferred three times to wells containing immobilized deselection HLA/peptide targets and incubated for 20 min at 4°C. At the end of the deselection process, ternary ribosomal display complexes were used for selection on HLA-A∗0201/NY-ESO1_157-165_(9V).

#### Binding screening of DARPins to HLA-A∗0201/NY-ESO1_157-165_(9V) using homogenous time-resolved FRET (HTRF)

DARPin clones selected by ribosome display were cloned into a derivative of the pQE30 (Qiagen) expression vector and transformed into *E. coli* XL1-Blue (Stratagene). Transformants were plated on LB agar with ampicillin and incubated overnight at 37°C. Single colonies were picked into individual wells of twelve 96-well plates containing 165 μL growth medium (LB containing 1% glucose and 50 μg/mL ampicillin) and grown overnight at 37°C with shaking at 800 rpm. Fresh LB medium (150 μL, 50 μg/mL ampicillin) was inoculated with 8.5 μL of the overnight culture, and protein expression was induced with isopropyl β-D-1-thiogalactopyranoside (IPTG) (0.5 mM) for 6 h at 37°C. Cells were harvested by centrifugation, resuspended in 8.5 μL B-PERII (Thermo Scientific) and incubated for 1h at RT with shaking at 600 rpm. Then, 160 μL PBS was added, and cell debris removed by centrifugation. The lysates were diluted 1:200 in PBSTB (PBS, 0.1% Tween 20, 0.2% [w/v] BSA, pH 7.4) containing 20 nM biotinylated target, 1:400 anti-6His-D2 HTRF antibody (Cisbio, France), and 1:400 anti-strep-Tb (Cisbio, France) in a 384-well format. After a 120 min incubation at 4°C, plates were read on a Tecan M1000 instrument using standard HTRF settings. Lysates from each clone were tested for binding to biotinylated HLA-A∗0201/NY-ESO1_157-165_(9V) and to the negative controls HLA-A∗0201/NY-ESO1_157-165_(9VAA) and HLA-A∗0201/EBNA-1. The specificity of each DARPin was evaluated by calculating the ratio of the HTRF signal obtained for HLA-A∗0201/NY-ESO1_157-165_(9V) versus the two negative controls. All DARPin binders that produced at least 25-fold higher HTRF signals with HLA-A∗0201/NY-ESO1_157-165_(9V) than with the negative controls were designated as specific preliminary hits for further characterizations.

#### Production of DARPin candidates

The 26 top DARPin candidates identified for HLA-A∗0201/NY-ESO1_157–165(9V) were purified using immobilized metal affinity chromatography (IMAC) in 96-well format and subsequently re-buffered into PBS. A single colony for each DARPin was grown overnight at 37 °C in TB medium containing 1% glucose and 50 μg/mL ampicillin, shaking at 700 rpm. Fresh TB medium (50 μg/mL ampicillin) was then inoculated 1:10 with the overnight culture and incubated at 37 °C at 700 rpm. After 2 h, IPTG (0.5 mM) was added to induce protein expression for 6 h at 37 °C. Cells were harvested by centrifugation (3,200 × g, 6 min), then lysed by incubation with B-PER II (Thermo Fisher), DNase I (200 U/mL), and lysozyme (0.4 mg/mL) for 60 min at room temperature, shaking at 900 rpm. Cell debris was removed by centrifugation (3,200 × g, 60 min, 4 °C), and the 0.65 μm–filtered supernatant was purified via IMAC (HisPur Cobalt Spin Plates, Thermo Fisher) through the N-terminal His tag. Washing steps were performed, followed by elution with 150 mM imidazole. Finally, DARPin molecules were re-buffered into PBS using Zeba Spin Desalting Plates (Thermo Scientific).

#### Surface plasmon resonance assessment of binding of DARPin binding to HLA/peptide targets

An inverse ProteOn setup was used to preliminarily test the interactions of each DARPin candidate with HLA-A∗0201/NY-ESO1_157-165_(9V). An RGS-His antibody (Qiagen ID 34650) was immobilized on a GLC chip to capture MRGS-6His tagged DARPin as ligands. HLA-A∗0201/peptide targets were immobilized as analytes on a neutravidin chip at different dilutions in a HEPES buffer.

#### Design and optimization of DARPin T cell engagers using a selection of anti-CD3ε−specific DARPins

Three different variants of the anti-CD3ε-specific DARPin domain of the bispecific T cell engager were tested. These variants resulted from affinity maturations (data not shown) and were used to identify the most suitable affinity for T cell engagement. Sequences and point mutations introduced into these three variants are described in Table S1.

#### Design of linkers for optimization of the bispecific DARPin T cell engagers

Different peptide linkers, varying in lengths and composition, were introduced between the HLA-A∗0201/NY-ESO1_157-165_(9V)-binding DARPins and CD3ε-binding DARPin domains. Five different linker lengths were tested, including XXS (GSPTGS), XS (GSPTPTPTTGS), S (GSPTPTPTTPTPTPTTGS), M (GSPTPTPTTPTPTPTTPTPTPTGS), and L (GSPTPTPTTPTPTPTTPTPTPTTPTPTPTTPTPTPTGS).

#### Size exclusion chromatography (SEC) analyses

DARPin molecules were loaded at 2 mg/mL onto an analytical SEC column (Superdex 200, GE Healthcare) in PBS buffer and examined for monodispersity, aggregation, and oligomerization.

#### Circular dichroism measurements

Circular dichroism was performed using a Jasco J-815 instrument and a 1 cm pathlength cuvette (Hellma). The molar residue ellipticity at 222 nm was recorded from 20°C to 90°C. Spectra from 190 to 250 nm were measured before and after the temperature measurement at 20°C. Samples were prepared at 1 μM in PBS.

#### Alanine scanning mutagenesis assays

Each residue of NY-ESO1_157-165_(9V) was sequentially substituted with alanine, except for anchoring positions p2 and p9, which were mutated simultaneously. T2 cells pulsed with 10^−6^ M of each alanine substituted peptide were incubated with effector CD8^+^ T cells for 4 h in the presence of DARPin candidates at concentrations corresponding to the EC_90_ levels for NY-ESO1_157-165_. Intracellular IFNγ in CD8^+^ T cells was detected by flow cytometry using anti-human IFNγ antibody (BD, 554702).

#### X-scanning mutagenesis assays

Each peptide position in NY-ESO1_157-165_(9V) was mutated to all 19 other amino acids. T2 cells were pulsed with each of the mutated peptides and incubated for 4 h with effector CD8^+^ T cells in the presence of DARPin candidate at an EC90 concentration, relative to wild type peptide. All experiments were performed in duplicates and repeated independently twice. The data was averaged and normalized to 100% for the for the corresponding wild-type residue at each position. A threshold of 30% T cell activation was used to identify potential amino acid substitutions tolerated by NY_1 and NY_2. These data were used to generate peptide motifs for NY_1 ([ACFGHILMNQRSTVWY]-[ACILMQV]-[ACDFGHILMNPQSTVW]-[FILMT]-W-[FILMV]-[CFILMQRSTVY]-[QT]-[ACITV]) and NY_2 ([ACFHIMNPQRSTVWY]-[FILMVW]-[ILMQV]-[IM]-W-[FILMPVW]-[ACFGILMNQRSTV]-[ACDEFGHKLMNPQRSTVWY]-[ACFGILSTV]), which were scanned against the SwissProt database^80^ taxonomically filtered to *Homo sapiens* using ScanProSite.^81^ All potentially cross-reactive epitopes (Table S2) were synthesized (GenScript, Piscataway, NJ, USA) and tested for their ability to mediate CD8^+^ T cell activation in the presence of pulsed T2 cells and each DARPin T cell engager.

#### T cell activation assays using T2 cells

T2 cells were pulsed overnight at 37°C with 10 μM of the NY-ESO1_157-165_(9V) peptide in serum free medium. Unpulsed T2 cells served as control. Effector CD8^+^ T cells were added at an effector to target ratio 1:5 in the presence of DARPins at the indicated concentrations, and incubated for 4–5 h at 37°C in the presence of Golgi-plug (Cat No. 555029, BD Biosciences). Afterward, cells were washed and stain with Live/Dead for 30 min at 4°C. Cells were then permeabilized overnight at 4°C using Cytofix/Cytoperm buffer (Cat No. 554722, BD Biosciences). The antibody against IFN-γ (BD, 554702) was then added in permeabilization buffer for 30 min at 4°C to stain for Intracellular IFN-γ. Cells were washed and resuspended in PBS before acquisition by FACS.

#### T cell activation assays using tumor cell lines

HLA-A∗0201^+^/NY-ESO1^+^ and HLA-A∗0201^+^/NY-ESO1^-^ tumor cells were incubated with PBMCs at an effector-to-target ratio of 10:1 for 48 h at 37°C. Activation was monitored by measuring the expression of the surface markers CD25 and CD69 on CD8^+^ T cells via flow cytometry. Supernatants were collected for IFNγ quantification using the Luminex technology. Antibodies used for flow cytometry analysis are listed in Table S6.

#### Chromium release cytotoxicity assays

Cellular cytotoxicity was evaluated using a standard 4-h ^51^Cr release assay (PerkinElmer) set up in triplicate in round-bottom 96-well plates. Briefly, HLA-A*∗0201*^*+*^*/NY-ESO1*^*+*^ *or HLA-A∗*0201^+^/NY-ESO1^–^ target cells, as well as peptide-pulsed or unpulsed T2 cells, were labeled by incubation with 10 mCi/mL ^51^Cr for 1 h, washed, and then seeded at 1,000 cells per well in 50 μL of complete T cell medium. Next, 100 μL of CD8^+^ T cells was added to each well at a 10:1 effector-to-target ratio, along with varying concentrations of DARPin molecules. After 4 h at 37 °C, supernatants were collected, and radioactivity was measured. The percentage of specific lysis was calculated according to the formula: % specific lysis = ((experimental release - spontaneous release)/(maximum release - spontaneous release)) x 100.

#### Crystal structure of NY_1

While screening for crystals of the NY_1/HLA-A∗0201/NY-ESO1_157-165_(9V) complex (concentrated to ∼10 mg/mL in 20 mM HEPES, 10% glycerol, 300 mM NaCl pH 7.5), we obtained a dataset of NY_1 alone at 1.8 Å resolution. Crystallization was conducted at the Protein Science Facility at Karolinska Institutet (http://ki.se/psf). Single crystals of NY_1 were obtained in condition 1–48 (pH 8.5; 0.12 M alcohol mix; 0.1 M buffer system 3; 37.5% (v/v) MPD_P1K_P3350) of the Morpheus screen (Molecular Dimensions Ltd.). The dataset was collected at a wavelength of 0.96880 Å at the I24 microfocus beamline at the Diamond synchrotron light source (DLS, UK). The structure was solved by molecular replacement using the previously determined crystal structure of another DARPin (PDB: 2XEH). The model^82^ was re-built and refined iteratively using Coot^83^ and Phenix^84^ to final R and R_free_ values of 18.3 and 21.5%, respectively (Table S5).

#### Cryo-electron sample preparation and data acquisition

The Avi-tag of HLA-A∗0201 was substituted by Strep-tag-II (STII) applying sequence and ligation independent cloning (SLIC).^85^ SLIC was also applied to introduce a Tobacco Etch Virus protease (TEV) cleavage site following the N-terminal His tag of DARPin NY_1. Refolded HLA-A∗0201-STII/NY-ESO1_157-165_(9V) was obtained as described above but purified further through Strep-Tactin Superflow high-capacity columns (1 mL, IBA lifescience) run in 20 mM HEPES, 300 mM NaCl pH 7.5. After a column wash, the protein was eluted using the same buffer supplemented with 2.5 mM desthiobiotin. TEV-cleaved DARPin NY_1 was reverse-purified via IMAC and the monomer was isolated from Superdex 200 equilibrated in 20 mM HEPES, 150 mM NaCl pH 7.5. For cross-linking, HLA-A∗0201/NY-ESO1_157-165_(9V) was mixed in a 1:2 M ratio with NY_1, concentrated to an absorbance at 280 nm (Abs280) of 2.3, and incubated for 45 min in 25 mM HEPES, 150 mM NaCl, supplemented with 1.4 mM BS(PEG)5 (BS5) (Thermo Fisher Scientific). The cross-linker was quenched by addition of 25 mM Tris. Samples for grid screening were isolated from Superdex 200 GL 10/300 SEC in 25 mM HEPES, 150 mM NaCl, pH 7.4, and concentrated to Abs280 values of ≥2 (Figure S12).

UltrAuFoil holey grids (R 0.6/1 gold foil on gold 300 mesh; Quantifoil Micro Tools GmbH) were glow-discharged for 60 s at 20 mA using a Glo-Qube (Quorum) instrument. Purified NY_1/HLA-A∗0201/NY-ESO1_157-165_(9V) complexes were thawed, centrifuged (14,000 g for 5 min at 4°C) and diluted to ∼0.5 mg/mL with gel filtration buffer. Protein was loaded into the freshly glow-discharged grids and plunge-frozen in LN_2_-cooled liquid ethane using a Vitrobot Mark IV (Thermo Fisher Scientific) with a blot force of 0 for 4 s. Temperature and relative humidity were maintained at 4°C and 100%, respectively. Grids were clipped and loaded into a 300-kV Titan Krios G3i microscope (Thermo Fisher Scientific, EPU 2.8.1 software), equipped with a Gatan BioQuantum energy filter and a K3 Summit direct electron detector (AMETEK). Grids were screened for quality control based on particle distribution and density, and images from the best grid were recorded. Micrographs were recorded at a nominal magnification of ×130,000, corresponding to a calibrated pixel size of 0.648 Å. The dose rate was 10.4 electron physical pixels per second, and images were recorded for 2.3 s divided into 45 frames, corresponding to a total dose of 57.5 electrons per Å^2^. The defocus range was set between −0.6 μm and −2.5 μm. Gain-corrected image data was acquired.

#### Cryo-EM data processing

The data processing workflow for the NY_1/HLA-A∗0201/NY-ESO1_157-165_(9V) dataset is illustrated in Figure S5. All image processing steps were performed using cryoSPARC v4.0.3.^86^ Movie stacks were motion-corrected, and the contrast transfer function (CTF) was estimated in the CryoSPARC Live Session, using Patch Motion Correction and Patch CTF estimation. Poor-quality micrographs were excluded based on a CTF fit threshold of 10 Å, leaving 7,781 micrographs for further processing. Particle picking began in CryoSPARC Live with blob-picker, and live 2D classification of approximately 100,000 particles yielded five ‘protein-like’ 2D classes (12,000 particles), which were used as templates for additional template picking. This process yielded approximately 7.7 million particles in total. A subset of 2D classes containing 100,000 particles was subjected to *ab initio* reconstruction without imposing symmetry (C1 symmetry), generating two initial models. Repeated 2D classification reduced the dataset to 205,000 particles, which were divided into three classes with heterogeneous 3D refinement, producing three nearly identical classes. All 205,000 particles were then used for homogenous 3D refinement, local CTF refinement, and subsequent non-homogenous refinement^87^ (Figure S5). Local estimated resolution revealed minimal variation from the overall of 3.0 Å. 3DFSC analysis revealed anisotropy along the Y-direction but confirmed an anisotropy-corrected global resolution estimate of 3.1 Å. Auto-sharpening and density-modification in Phenix enhanced the map (Figures S6–S8). Post-processing with DeepEMhancer^88^ did not yield further improvements in the DARPin/HLA-peptide interface region.

#### Cryo-EM model building and refinement

The crystal structures of NY_1 and HLA-A∗0201/NY-ESO1 (PDB: 1S9W)^89^ were placed into the initial map in ChimeraX.^90^ The model was iteratively refined by model building in Coot and ChimeraX-Isolde,^91^ followed by real-space refinement in Phenix using an integrated Amber force field with Ramachandran, secondary structure, and reference structure restraints.^83^^,^^91^^,^^92^^,^^93^ Post-processed maps were generated in cryoSPARC and Phenix, respectively.^45^^,^^88^^,^^94^ Progress in structure refinement and map quality was assessed using Fourier Shell correlation plots, map-model CC, EM-ringer score, and model geometry statistics in Phenix^92^ and Molprobity.^95^ Density modification algorithms had the greatest impact on the DARPin map, while the auto-sharpened HLA/peptide map was almost identical to its density-modified version.

#### Structural analysis and visualization

Structures were analyzed and visualized using ChimeraX^90^ and PyMOL (Schrödinger). Molecular interactions and buried surface areas were determined with the Arpeggio^96^ and PISA^97^ webservers. Void volumes and the predicted numbers of water molecule counts were obtained via CastP^98^ and Amber 3DRISM analyses.^52^^,^^53^ All results were organized and visualized using custom scripts primarily based on the R tidyverse and ggplot2 packages.

### Quantification and statistical analysis

The number of experiments (n) underlying each plot or analysis is indicated in the respective Figure legends. Dose-response curves (Figures 2A, 4A, 4C, and 5A–5D) were obtained by non-linear regression applying the three parameter log(agonist) vs. response model as implemented in Graph Pad Prism 10. Lines in jitter plots (Figures 4B, 4D, and 5E–5H) represent the mean.

### Additional resources

We have not produced any additional resources relevant to this study.
