# Supplementary material for: Development of DARPin T cell engagers for specific targeting of tumor-associated HLA/peptide complexes
Source: iScience. 2025 Nov 3;28(12):113926. doi: 10.1016/j.isci.2025.113926 (PMC12664392; doi:10.1016/j.isci.2025.113926)
Supplement: Document S1. Figures S1–S12 and Tables S1–S7 [file mmc1.pdf]

iScience, Volume *28*

Supplemental information

Development of DARPin T cell engagers for specific targeting of tumor-associated HLA/peptide complexes

Natalia Venetz-Arenas, Tim Schulte, Sandra Müller, Karin Wallden, Stefanie Fischer, Tom Resink, Nadir Kadri, Maria Paladino, Nicole Pina, Filip Radom, Denis Villemagne, Sandra Bruckmaier, Andreas Cornelius, Tanja Hospodarsch, Evren Alici, Hans-Gustaf Ljunggren, Benedict J. Chambers, Xiao Han, Renhua Sun, Marta Carroni, Victor Levitsky, Tatyana Sandalova, Marcel Walser, and Adnane Achour

Supplemental Figures and Tables

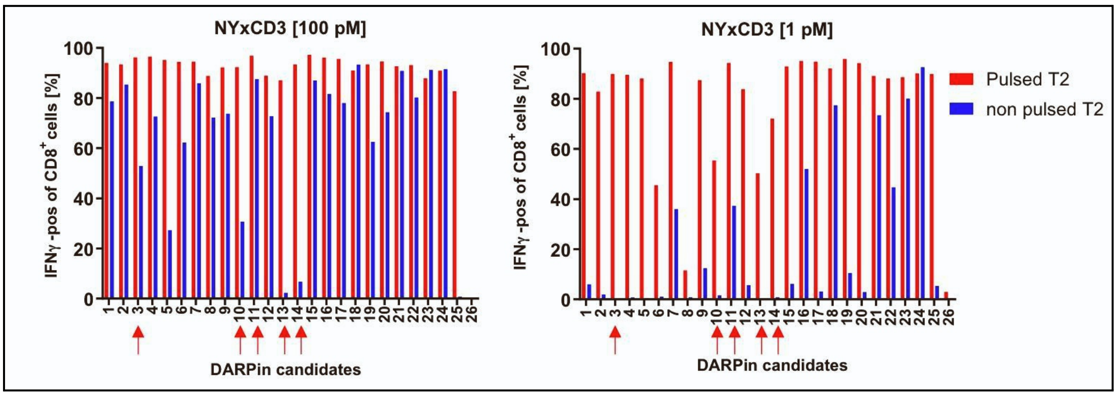

Figure S1. Functional screening of DARPin TCEs using NY-ESO1$_{157\text{-}165}$(9V)-pulsed T2 cells. Related to Figure 1.

26 different DARPin TCEs were tested for their capacity to elicit intracellular IFNγ release in CD8+ T cells in the presence of T2 cells pulsed with 1 and 100 pM NY-ESO1157-165(9V). The effects of peptide-pulsed T2 cells (red) on IFNγ production by CD8+ T cells were compared to the effects of non-pulsed (blue) T2 cells. DARPin TCEs that provoked significant IFNγ release by CD8^{+} T cells in the presence of peptide-pulsed T2 cells, and none in the presence of non-pulsed T2 cells were selected for further studies (indicated by red arrows).

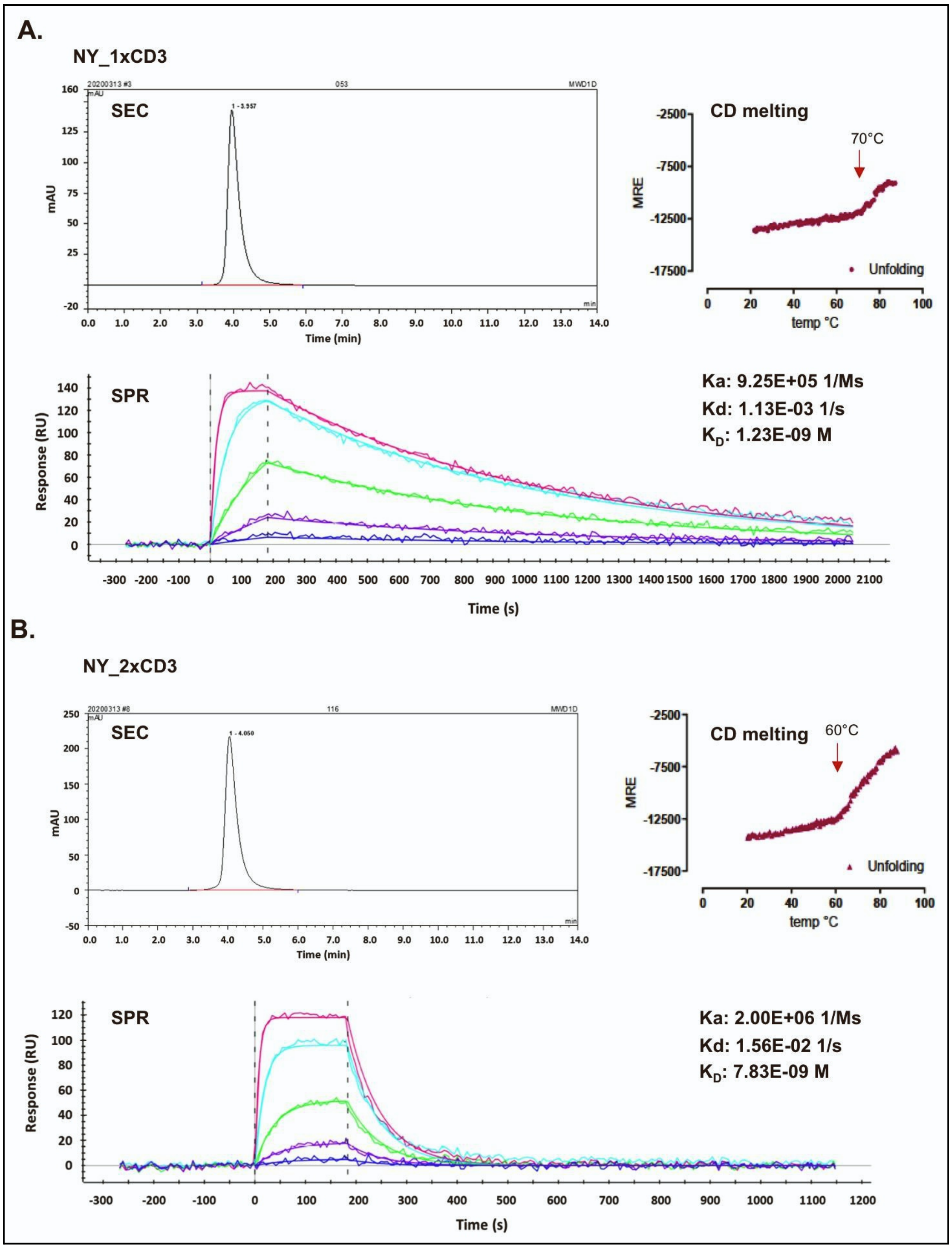

Figure S2. Biophysical characterization of the lead DARPin TCEs NY_1xCD3 and NY_2xCD3. Related to Figure 1.

Biophysical analyses of **A.** NY_1xCD3 and **B.** NY_2xCD3 characterize both DARPin TCEs as homogenous, stable and with high affinity to HLA-A*0201/NY-ESO-1$_{157\text{-}165}$(9V). Size exclusion chromatography (SEC) reveals single peaks, indicating homogeneous populations. Circular dichroism (CD) melting curves demonstrate that the two TCEs display high stability with T_m values at 70°C and 60°C for NY_1xCD3 and NY_2xCD3, respectively. Surface plasmon resonance

(SPR) analyses show similar nanomolar affinities to HLA-A*0201^{+}/NY-ESO1$_{157\text{-}165}$(9V), but different kinetics.

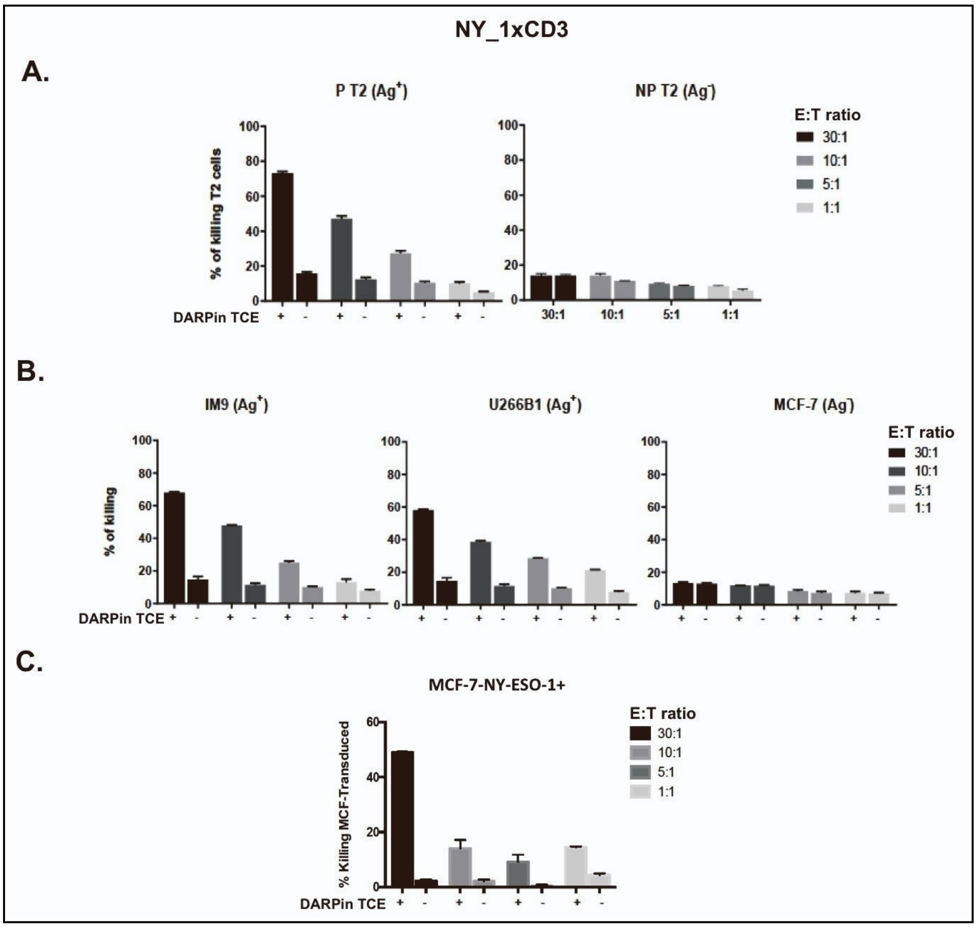

Figure S3. NY_1xCD3 mediates highly specific and efficient cytotoxicity towards HLA-A*0201^{+}/NY-ESO1$_{157\text{-}165}^{+}$ tumor cell lines. Related to Figure 2.

A. NY_1xCD3 enhances significantly the killing of T2 cells pulsed with 1 µM NY-ESO1$_{157\text{-}165}$ by endogenous CD8^{+} T cells. The addition or absence of NY_1xCD3 (10 nM) is indicated by + and -, respectively. E:T ratios stand for CD8 T cell effector:target cells. The percentage of specific lysis obtained for the different tumor cell lines by the chromium release assay is presented.

B. NY_1xCD3 provokes significantly more efficient killing of HLA-A*0201^{+}/NY-ESO1$_{157\text{-}165}^{+}$ but not HLA-A*0201^{+}/NY-ESO1$_{157\text{-}165}^{-}$ cancer cell lines.

C. NY_1xCD3 provokes efficient killing of the HLA-A*0201^{+}/NY-ESO1$_{157\text{-}165}^{-}$ cancer cell line MCF-7 following transfection with full-length NY-ESO1 molecule.

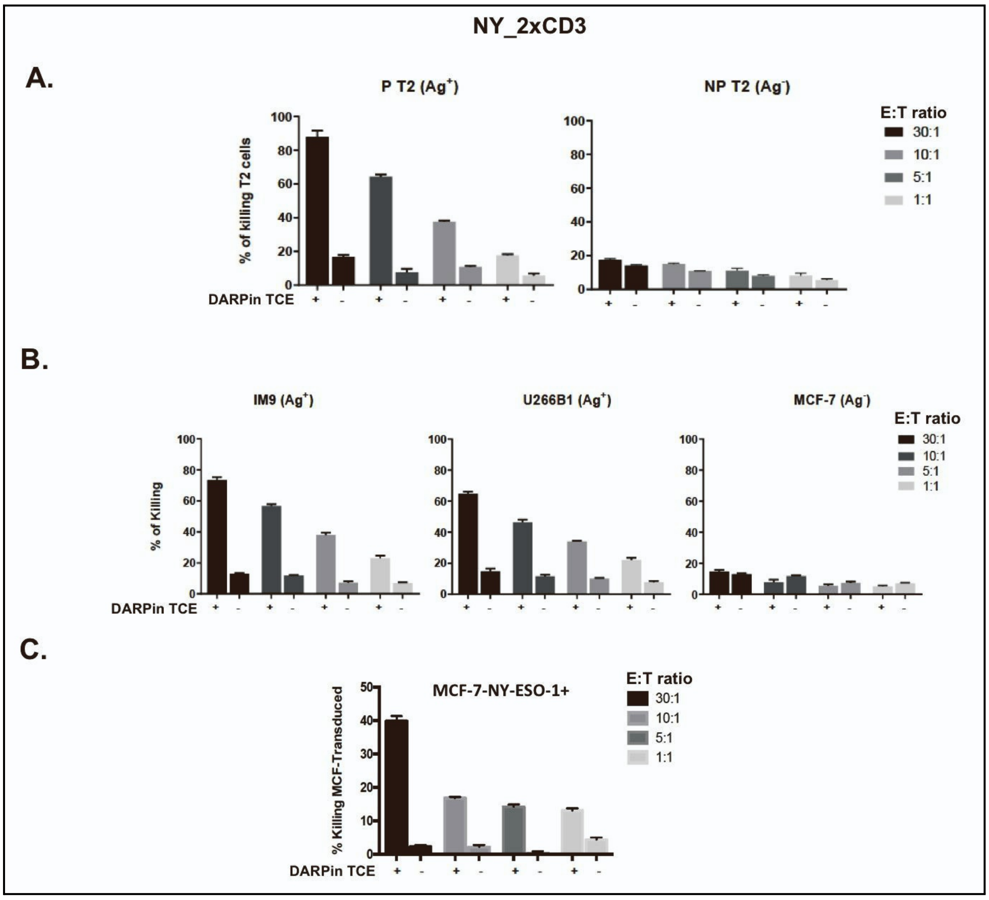

Figure S4. NY_2xCD3 mediates highly specific and efficient cytotoxicity towards HLA-A*0201^{+}/NY-ESO1$_{157\text{-}165}^{+}$ tumor cell lines. Related to Figure 2.

A. NY_2xCD3 enhances significantly the killing of T2 cells pulsed with 1 µM NY-ESO1$_{157\text{-}165}$ by endogenous CD8^{+} T cells. The addition or not of NY_1xCD3 (10 nM) is indicated by + and -, respectively. E:T ratios stand for CD8 T cell effector:target cells. The percentage of specific lysis obtained for the different tumor cell lines by the chromium release assay are shown.

B. NY_2xCD3 provokes significantly more efficient killing of HLA-A*0201^{+}/NY-ESO1$_{157\text{-}165}^{+}$ but not HLA-A*0201^{+}/NY-ESO1$_{157\text{-}165}^{-}$ cancer cell lines.

C. NY_2xCD3 provoke efficient killing of the HLA-A*0201^{+}/NY-ESO1$_{157\text{-}165}^{-}$ cancer cell line MCF-7 following transfection with full-length NY-ESO1 molecule.

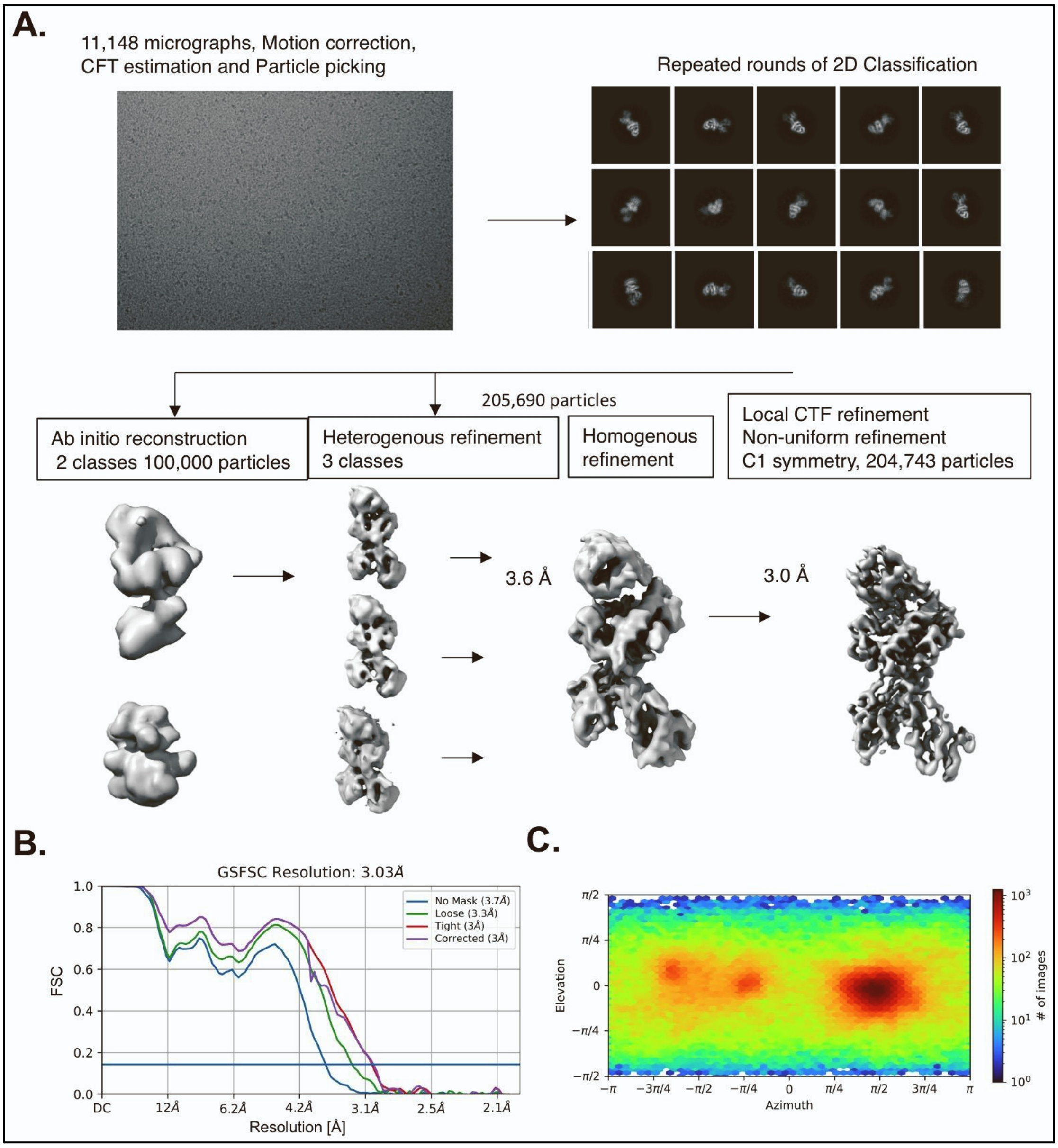

Figure S5. Cryo-EM processing scheme for the determination of the ternary NY_1/HLA-A*0201^{+}/NY-ESO1$_{157\text{-}165}$ structure. Related to Figure 6.

A. Cryo-EM data processing workflow.

B. Overall resolution estimation by Fourier Shell Correlation (FSC) with threshold 0.143.

C. Viewing direction distribution plot (obtained from cryoSPARC v3.2.0 [1]).

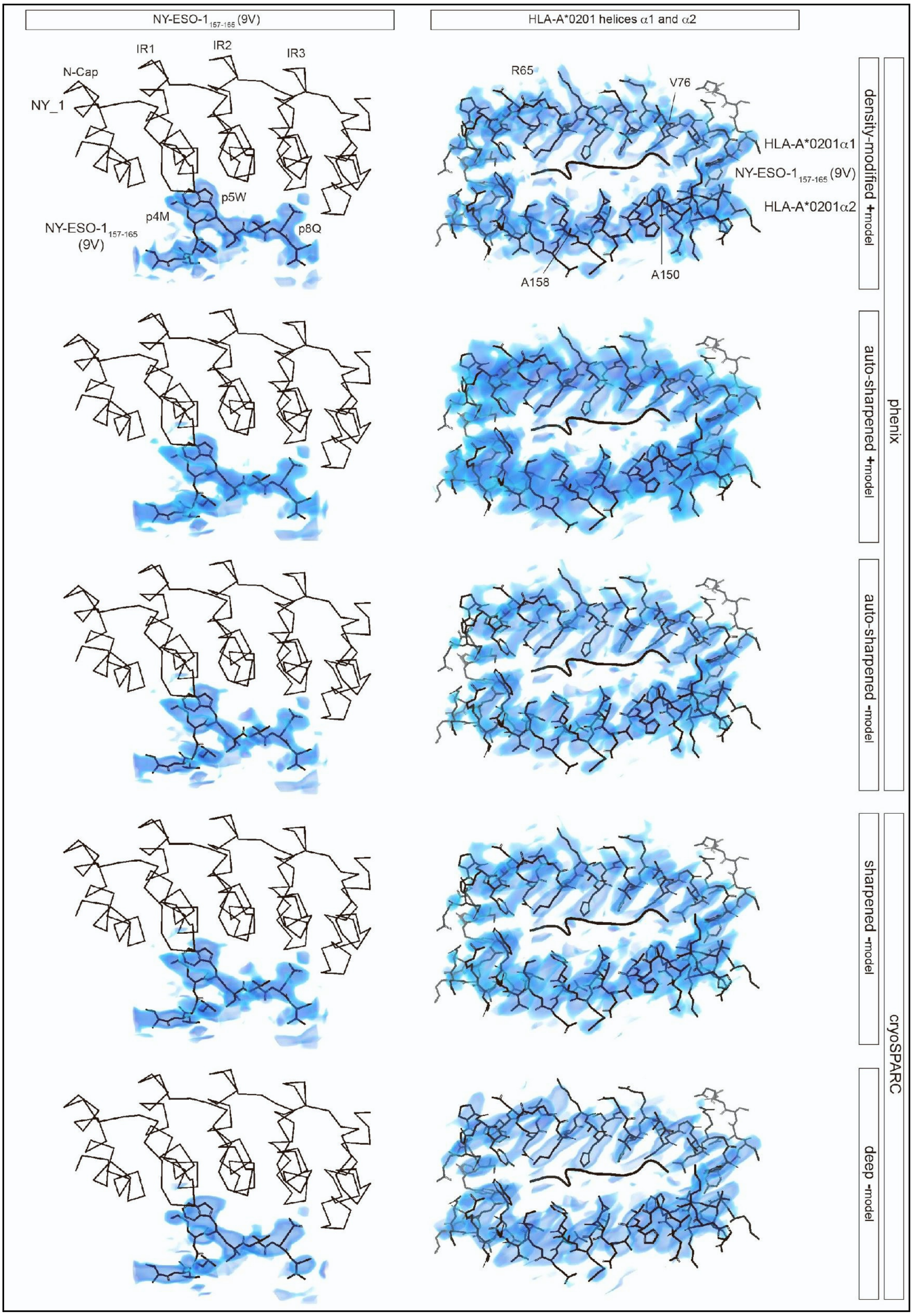

Figure S6. Map comparison of the regions defining the NY-ESO1$_{157\text{-}165}$ peptide and the HLA-A*0201 α-helices. Related to Figure 6.

Volumes of five post-processed maps are shown for the peptide NY-ESO1$_{157\text{-}165}$(9V) and the two HLA-A*0201 α-helices in the same orientations as in Figure 6D,

organized as row- and column-wise panels, respectively. From top to bottom, volumes were obtained after model-based density-modification (+), and auto-sharpening with (+) and without (-) model in Phenix as well as sharpening and deep-enhancement in cryoSPARC.[2–4] Volume color ramp is identical to that in Figure 6D. By comparing the maps of regions shown in Figures S6 and S7 and the model-map FSC profiles, we selected the density-modified map as the final map for presentation.

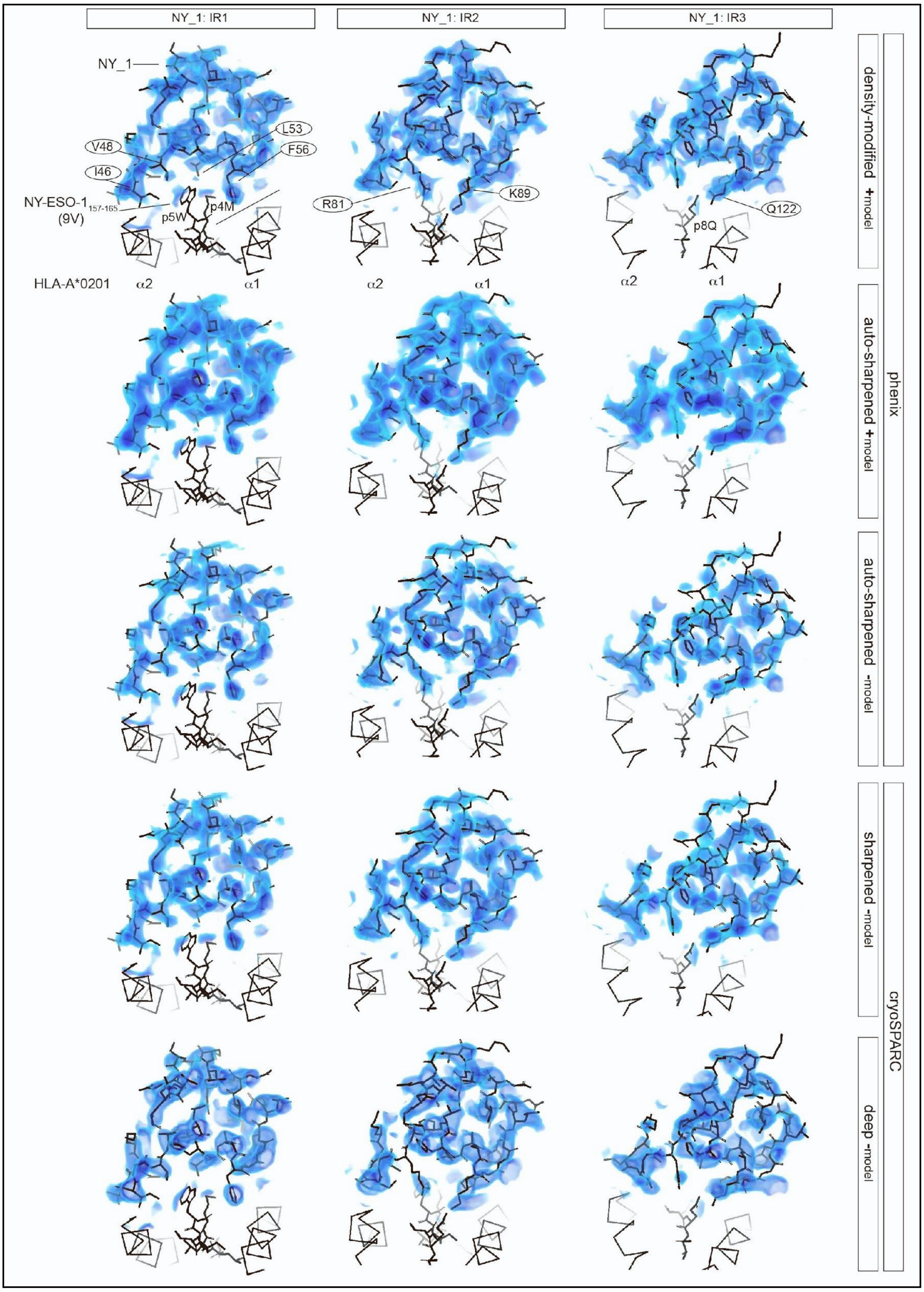

Figure S7. Map comparison of the regions defining internal repeats of NY_1. Related to Figure 6.

Volumes of five post-processed maps are shown for the three internal repeats of NY_1 in the same orientations as in Figure 6D, organized as row and column-wise panels, respectively. Volumes obtained as described in Figure S5.

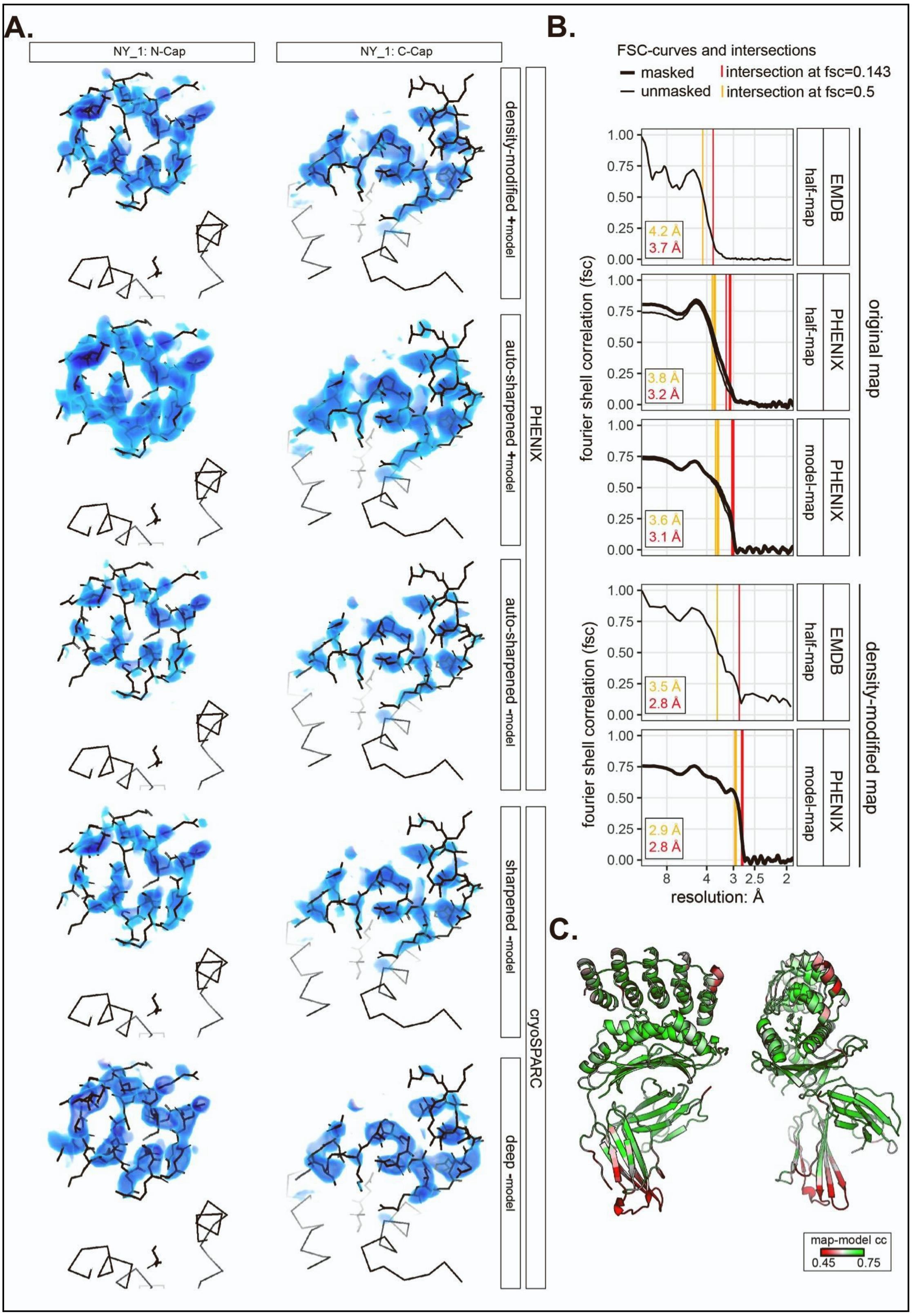
A.
NY_1: N-Cap
NY_1: C-Cap
density-modified +model
auto-sharpened +model
auto-sharpened -model
sharpened -model
deep -model
PHENIX
cryoSPARC
B.
FSC-curves and intersections
masked
unmasked
intersection at fsc=0.143
intersection at fsc=0.5
fourier shell correlation (fsc)
EMDB half-map
PHENIX half-map
PHENIX model-map
original map
density-modified map
4.2 Å
3.7 Å
3.8 Å
3.2 Å
3.6 Å
3.1 Å
3.5 Å
2.8 Å
2.9 Å
2.8 Å
resolution: Å
C.
map-model cc
0.45
0.75

Figure S8. Map comparison of the regions defining the cap elements of NY_1, as well as presentation of half-map and model-map FSC curves and map-model cross-correlation. Related to Figure 6.

A. Volumes of five post-processed maps are shown for the N- and C-cap of NY_1 in the same orientations as in Figure 6D, organized as row and column-wise panels, respectively. Volumes were obtained as described in Figure S5.

B. Half-map and model-map Fourier Shell Correlation (FSC) curves for the initial EMD-50336 deposited map and the five post-processed maps, respectively. The FSC curve of the masked half-maps crosses the value of 0.143 (red lines) at a resolution of 3.1 Å. For the five maps, the model-map FSC curves cross the value of 0.5 (orange lines) at resolutions of 2.9, 3.5, 3.5, 3.5 and 3.9 Å from top to bottom, respectively. The alternate model-based resolution estimate d_{model} [5] gives values of 2.9, 3.2, 3.4, 3.3 and 3.3 Å, respectively. Curves related to masked and unmasked maps are displayed as solid and dotted lines.

C. The structure is colored in red, white and green for map-model cross-correlation values of 0.45, 0.6 and 0.75, respectively. Map-model cross-correlation values of 0.5 and 0.7 indicate poor and good fits.[5] The views are identical to those in Figures 6A and 6B.

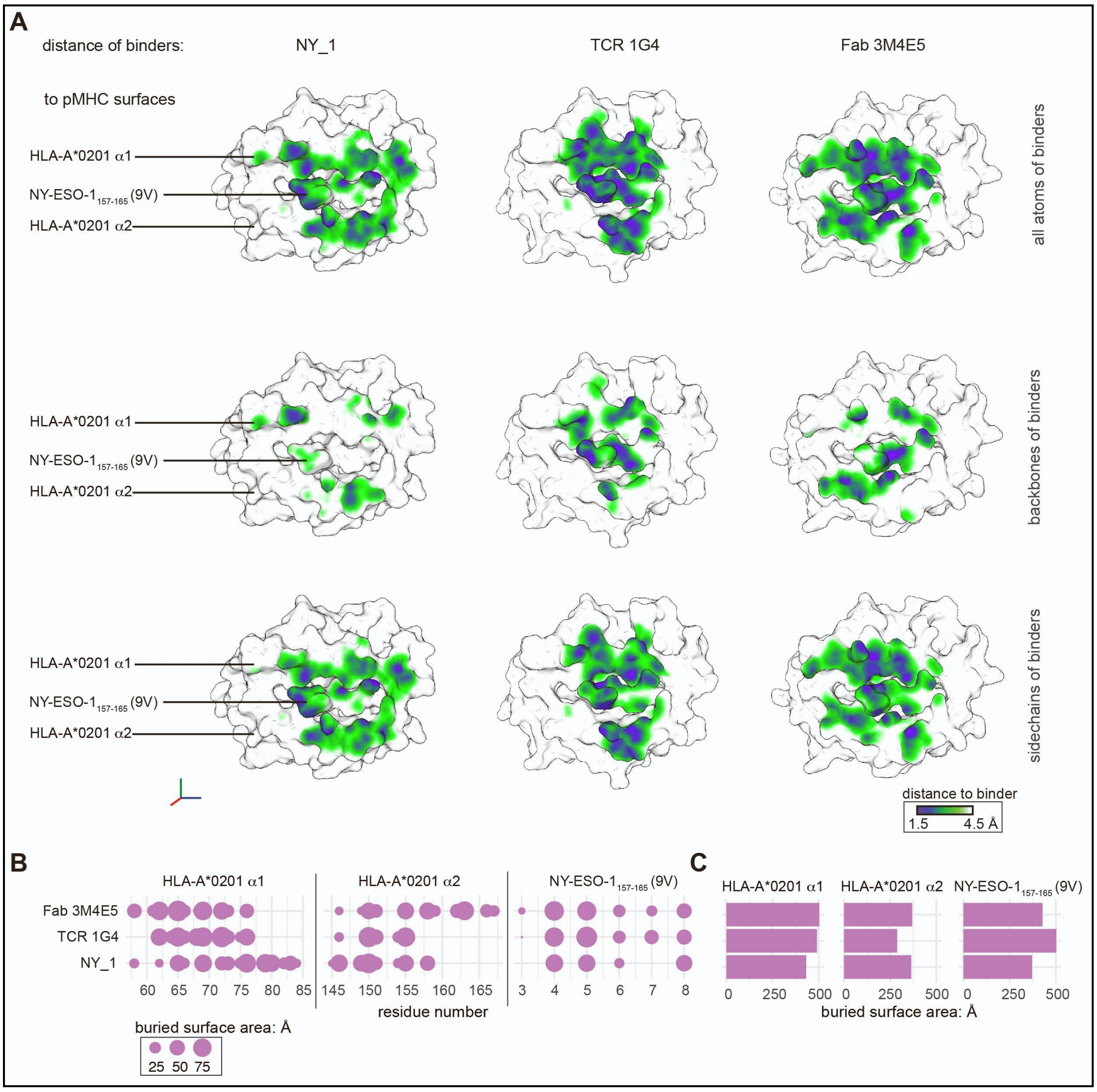

Figure S9. In contrast to NY_1, the TCR 1G4 and the Fab fragment 3M4E5 bind to HLA-A*0201/NYESO1$_{157\text{-}165}$(9V) with tighter interfaces and closer binder-to-target distances. Related to Figure 7.

A. The distance ramps between DARPin NY_1 (left panel), TCR 1G4 (middle) and Fab fragment 3M4E5 (right), and HLA-A*0201/NY-ESO1$_{157\text{-}165}$(9V) are presented for all atoms (top panel), backbone atoms (middle panel) and side chains (bottom panel). This structural comparison demonstrates that the CDRs of both 1G4 and 3M4E5 form tighter interfaces with the surface of HLA-A*0201/NY-ESO1$_{157\text{-}165}$(9V), compared to the binding mode and interface used by NY_1.

B. Residue-level buried surface areas were obtained from PISA [6] and plotted separately for each binder in panels for the HLA-A*0201 helices α1 and α2 as well as the NY-ESO1$_{157\text{-}165}$(9V) peptide. The areas of the filled circles scale with the buried surface area (Å^2).

C. The residue-level buried surface areas are summed for each binder and the panels of HLA-A*0201 helices α1 and α2 as well as NY-ESO1$_{157-165}$(9V).

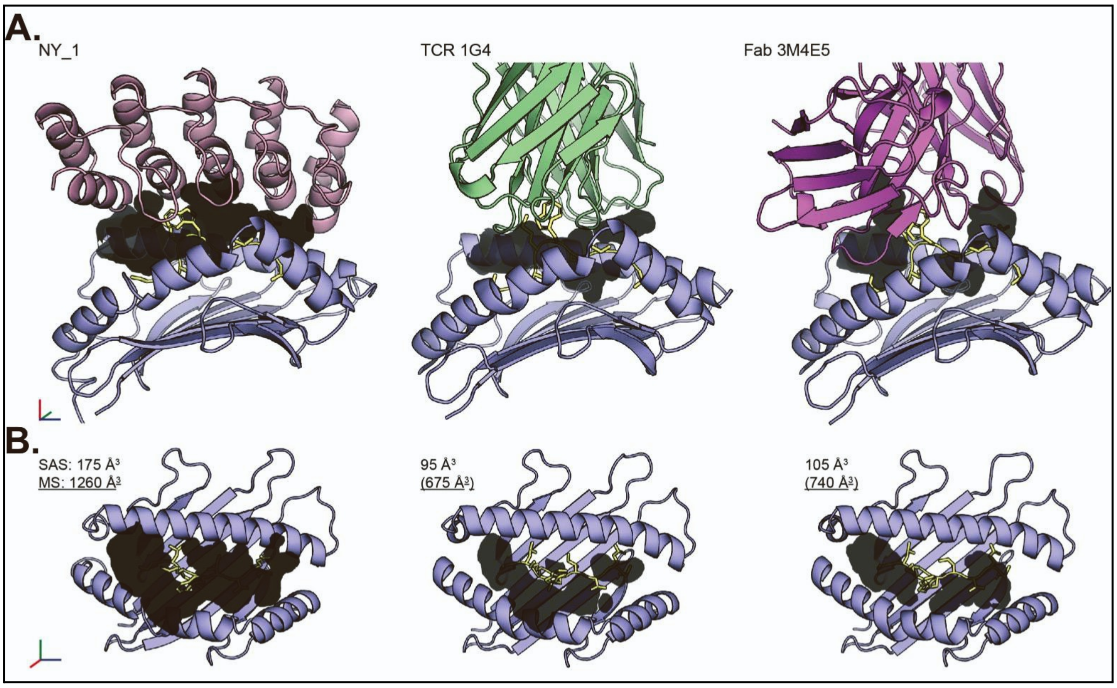

Figure S10. The NY_1/HLA-A0201/NYESO1$_{157-165}$(9V) interface comprises a significantly larger void compared to those formed with TCR 1G4 or Fab 3M4E5. Related to Figure 7.

A. Voids in contact with the peptide for complexes of HLA-A0201/NY-ESO1$_{157-165}$(9V) with DARPin NY_1, TCR 1G4 and Fab fragment 3M4E5 were obtained using CastP.[7]

B. The summed volumes based on solvent-accessible surfaces (SAS) molecular surfaces (MS) are listed besides each complex. The analyses were performed only for surfaces created by atoms localized within 8 Å around the peptide, and applying a default probe radius of 1.4 Å. The voids are shown in dark within both the side- and top-views.

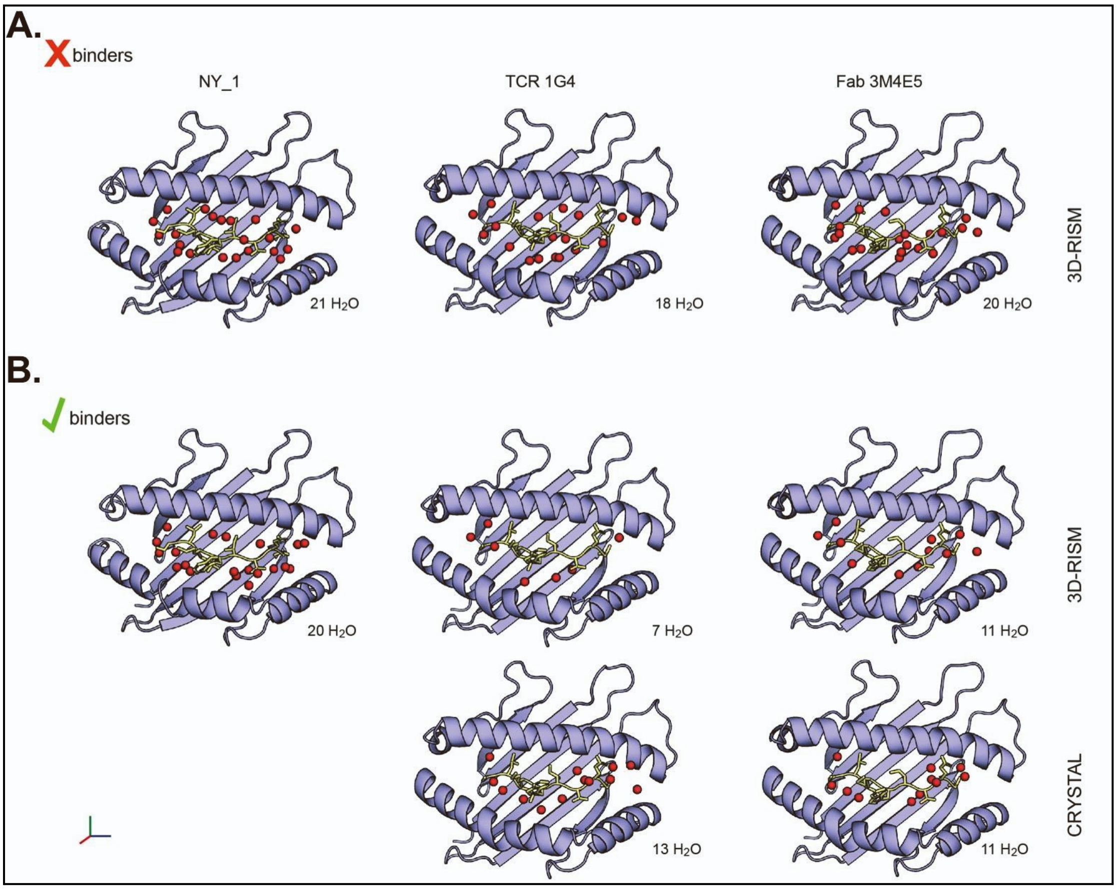

Figure S11. A larger amount of water molecules is predicted to remain within the NY_1/HLA-A*0201/NY-ESO1$_{157\text{-}165}$(9V) interface after binding to the DARPin molecule. Related to Figure 7.

A. Predictions using 3D-RISM [8] were made for HLA-A*0201/NY-ESO1$_{157\text{-}165}$(9V) prior to binding to NY_1, 1G4 and 3M4E5, revealing similar amounts of H_2O molecules surrounding the presented peptide.

B. 3D-RISM predictions of the position and amount of water molecules at the interfaces formed between HLA-A*0201/NY-ESO1$_{157\text{-}165}$(9V) and NY_1 (left), 1G4 (middle) and 3M4E5 (right) indicated that water molecules remain within the NY_1/HLA-A*0201/NY-ESO1$_{157\text{-}165}$(9V). Water molecules found within the interfaces of the 1G4/HLA-A*0201/NY-ESO1$_{157\text{-}165}$(9V) and 3M4E5/HLA-A*0201/NY-ESO1$_{157\text{-}165}$(9V) interfaces are displayed for comparison.

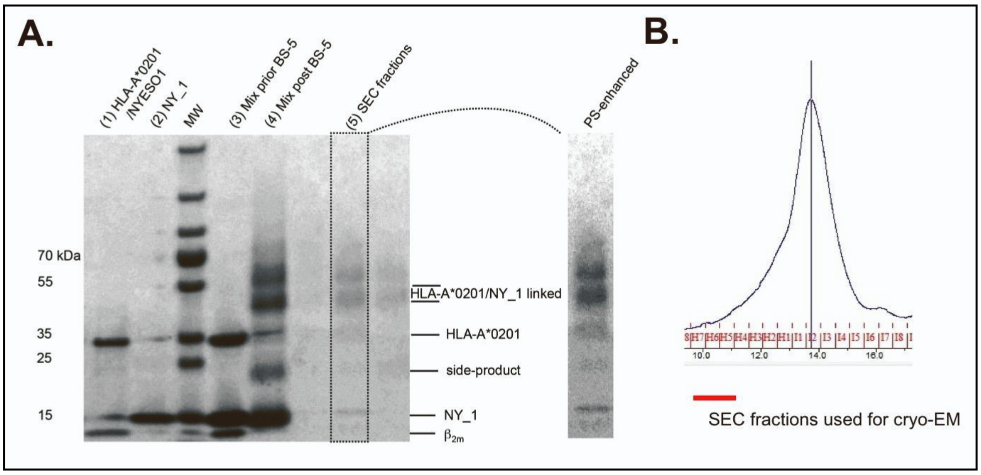

Supplementary Figure 12. Production and isolation of cross-linked DARPin NY_1/HLA-A*0201/NY-ESO1$_{157\text{-}165}$(9V) complexes

A. Sample preparation for collection of the high-resolution single particle cryo-EM dataset. The following samples were applied: (1) SEC and Streptactin-purified refolded HLA-A*0201/NY-ESO1$_{157\text{-}165}$(9V) (2) NiNTA- and SEC-purified NY_1 (3) 1:2 molar mix of HLA-A*0201/NY-ESO1$_{157\text{-}165}$(9V) and NY_1 prior (4) and post BS5 cross-linking, as well as the (5) final combined SEC-fractions used for cryo-EM. To make the bands in the fifth lane more visible, the selected region was cut out and its brightness and contrast levels were adjusted in Adobe Photoshop. Denaturing SDS-PAGE gradient gels were stained using silver blue.[9]

B. The cross-linked sample was purified by Superdex 200 10/300 to isolate the HLA-A*0201/NY-ESO1$_{157\text{-}165}$(9V)/NY_1 complex. The fraction corresponding to the main peak eluted after 14 mL and was concentrated for grid preparation.

Table S1. Amino acid sequences of the three CD3ε-specific DARPin variants used within this study. Related to Figure 2.

**CD3ε-specific DARPins**	**AA sequence** (N-cap/ IR1/ IR2/ C-cap)
version 1	DLGQKLLEAAWAGQDDEVRELLKAGADVNA
K_D: 35 ± 21 nM	KNSRGWTPLHTAAQTGHLEIFEVLLKAGADVNA
	KDDKGVTPLHLAAALGHLEIVEVLLKAGADVNA
	QDSWGTTPADLAAKYGHEDIAEVLQKAA
version 2	DLGQKLLEAAWAGQDDEVRELLKAGADVNA
K_D: 14 ± 3 nM	KNSRGWTPLHTAAQTGHLEIFEVLLKAGADVNA
	K**N**DK**R**VTPLHLAAALGHLEIVEVLLKAGADVNA
	**R**DSWGTTPADLAAKYGH**Q**DIAEVLQKAA
version 3	DLGQKLLEAAWAGQ**L**DEVR**I**LLKAGADVNA
K_D: 6 ± 0 nM	KNSRGWTPLHTAAQTGHLEIFEVLLKAGADVNA
	K**TN**K**R**VTPLHLAAALGHLEIVEVLLKAGADVNA
	**R**DTWGTTPADLAAKYGH**R**DIAEVLQKAA

DARPin amino acid sequences for the four repeats of CD3ε-specific DARPin versions 1-3.

Listed K_D-values were obtained by surface plasmon resonance.

Mutations identified during affinity maturation rounds are in red.

Table S2. Cross reactive peptides identified through X-scanning analyses of NY_1xCD3 and NY_2xCD3. Related to Figure 3.

NY_1xCD3				NY_2xCD3			
#	Sequence /%TCA	#		#	Sequence /%TCA)	#	Sequence /%TCA
01	SLLMWITQC /44%	41	VLLLWLLQV	01	SLLMWITQC /63%	41	RLVIWPGFT
02	AAGTWVLQA	42	WVSFWISQA	02	SLLMWLTPL /63%	42	RVLIWFISI
03	ACGIWMITV	43	YLNMWITTC	03	TLLIWLFEV /7%	43	RVLIWLINI
04	AMLLWVQQA	44		04	AIVIWFTGF	44	RWVMWFGDG
05	AQLLWFLQT	45		05	AIVIWIILA	45	SFLIWLLDF
06	AVFLWLVTI	46		06	ALVIWWQRV	46	SFLIWLLLC
07	AVLTWLSQT	47		07	AMVIWINEI	47	SIIIWVVWI
08	CVATWVFTA	48		08	CFIMWVLFI	48	SLLIWVISL
09	FIDMWFSTV	49		09	CIIIWLLAG	49	SLLMWMLRL
10	FLTLWLTQV	50		10	CIVMWLAGG	50	SLMIWLQTF
11	GVTTWIQTV	51		11	CLLIWLLDA	51	SLVIWICLV
12	HCLFWLLQV	52		12	CLMIWLIFS	52	SMVIWLLGF
13	HIGTWFTTT	53		13	CMVIWVLAF	53	SVVIWWIVC
14	HISTWLYQA	54		14	CVLIWVVGG	54	TFLMWFIET
15	ICLIWLLTV	55		15	FLIIWLITG	55	TIIIWLFFL
16	ICVIWLYTA	56		16	FLVIWILFS	56	TLQIWLRQA
17	LIQFWMSTV	57		17	FLVIWLVGF	57	TLQIWVIWL
18	LLGTWVFQV	58		18	HLVIWLLLV	58	TVLMWPRKI
19	LLPLWLSTT	59		19	IFLIWLLDF	59	TVMMWPLAV
20	LVITWIMTV	60		20	ILMIWLMAT	60	TVVMWVSAS
21	NLFLWLSTV	61		21	IMIIWVLAI	61	VILIWISVL
22	NLLIWVTTI	62		22	IVIIWIVSC	62	VLLIWLLTL
23	QISLWIITA	63		23	IVLIWVIAC	63	VLLMWLLVL
24	QLSMWIRTC	64		24	IVLIWVIAC	64	VVIIWLFLA
25	QMPLWVRQI	65		25	IVLIWVVSV	65	VVVMWIILA
26	QQAMWMMTA	66		26	IVVIWVIVS	66	VWLIWFTGS
27	RLCIWLLQT	67		27	MFIMWFSGL	67	VWVMWMRGG
28	RVMLWVTTA	68		28	MLVIWILTL	68	WLLIWLLLG
29	SLFLWIRTA	69		29	NIQIWLANG	69	
30	SLSTWIVTV	70		30	NIVIWVSGS	70	
31	SQCMWLMQA	71		31	NLLIWVTTI	71	

32	TIHTWIRQC	72		32	NLVIWPSVA	72	
33	TLGLWLTTA	73		33	NVLIWPMEG	73	
34	TLGLWMVTA	74		34	NVLIWPTDG	74	
35	TLLLWLCQA	75		35	PLLMWLLKS	75	
36	TLPIWMMQT	76		36	PLMIWVTDT	76	
37	TLQIWLRQA	77		37	PLVMWLQGG	77	
38	TLQTWLVQA	78		38	PVLMWVQAL	78	
39	VADTWVLTA	79		39	RLIIWILYL	79	
40	VAPLWMRQI	80		40	RLQIWPGYA	80	

List of potential cross-reactive peptides identified in the X-scanning for NY_1 and NY_2 DARPin candidates. T2 cells were pulsed with each of the peptides listed in the table and $CD8^+$ T cell activation (TCA) was evaluated (percentage intracellular IFN-γ, normalized to TCA of NY-ESO1$_{157–165}$(9V)-pulsed T2 cells). Both DARPin candidates were tested on the full set of peptides identified. In the absence of TCA, no percentage value is given in the table. Green font: TCA for naturally occurring NY-ESO1$_{157–165}$ peptide (SLLMWITQC). Red font: TCA in the range of the NY-ESO1$_{157–165}$ peptide. Orange font: low level of TCA.

Table S3. EC_{50} values for different TCEs. Related to Figure 4.

DARPin variant		EC_{50} [nM] NY_1xCD3	EC_{50} [nM] NY_2xCD3
Linker variant (length)	L (38 AA)	1.2	2.9
	M (24 AA)	0.4	1.2
	S (18 AA)	0.4	1.1
	XS (11 AA)	0.3	0.4
	XXS (6 AA)	0.1	0.3
CD3ε-binder variant	variant-1 (v1)	7.1	19.9
	variant-2 (v2)	0.5	0.7
	variant-3 (v3)	0.005	0.02

The EC_{50} values were obtained from the analysis of $CD8^+$ T cell activation curves for DARPin TCEs with varying linker lengths and CD3ε-binder variants (**Figures 4A and 4C**).

Table S4. Cryo-EM data collection, refinement and validation of the NY_1/HLA-A*0201/NY-ESO1$_{157-165}$(9V) complex. Related to Figure 6.

EMDB ID	EMD-50336
PDB ID	9FE1
**Data collection and processing**	
Magnification	130k
Voltage (kV)	300
Electron exposure (e–/Å^2)	57.5
Defocus range (μm)	-0.6 to -2.5
Pixel size (Å)	0.648
Symmetry imposed	C1
Initial particle images (no.)	7,726,585
Final particle images (no.)	204,743
Half-map based resolution (Å) at FSC$_{0.143}$	3.0
3DFSC global resolution (Å) / sphericity	3.1 / 0.74
**Refinement**	
Initial models used (PDB code)	9EPA and 1S9W
Map sharpening B_{iso} factor (Å^2)	143.4
Model composition	
Non-hydrogen atoms	8464
Protein residues	539
Ligands	0
Waters	0
B factors (Å^2)	
Protein (min / max / mean)	23.55 / 112.13 / 43.78
R.m.s. deviations	
Bond lengths (Å)	0.005
Bond angles (°)	0.849
Validation	
MolProbity score	1.82
Clashscore	9.57
Poor rotamers (%)	0.22
Ramachandran plot	
Favored (%)	95.48
Allowed (%)	4.52

Disallowed (%)	0.00
**Map-model correlation and resolution estimates**	sharpened / density-mod
$dFSC_{model}$ (Å)	3.5 / 2.9
d_{model} (Å)	3.2 / 2.9
CC_{mask} (0.5-0.7 for low-high correlation)	0.70 / 0.68
CC_{box}	0.77 / 0.58
CC_{peaks}	0.67 / 0.58
CC_{volume}	0.72 / 0.67

Table S5. Statistics of the crystal structure of DARPin NY_1. Related to Figure 6.

PDB ID	9EPA
**Data collection and processing**	
Resolution range	28.48 - 1.761 (1.824 - 1.761)
Space group	C 1 2 1
Unit cell	106.558 43.284 32.409 90 93.784 90
Total reflections	95069 (8028)
Unique reflections	14698 (1438)
Multiplicity	6.5 (5.6)
Completeness (%)	99.55 (96.77)
Mean I/sigma(I)	6.53 (2.17)
Wilson B-factor	18.22
R-merge	0.2024 (0.6391)
R-meas	0.2204 (0.7047)
R-pim	0.08609 (0.2918)
CC1/2	0.982 (0.796)
**Refinement**	
Reflections used in refinement	14691 (1437)
Reflections used for R-free	1460 (146)
R-work	0.1835 (0.2392)
R-free	0.2156 (0.2940)
CC(work)	0.960 (0.889)
CC(free)	0.971 (0.845)
Number of non-hydrogen atoms	1276
macromolecules	1154
ligands	0
solvent	122
Protein residues	157
RMS bonds/angles	0.007 Å / 0.93°
Ramachandran favored/allowed (%)	98.71/1.29
Ramachandran outliers (%)	0.00
Rotamer outliers (%)	0.00
Clashscore	3.40
Average B-factor	20.21
macromolecules	19.31
solvent	28.71

Table S6. Antibodies used for flow cytometry analyses. Related Star methods section "T cell activation assays using tumor cell lines".

Antibody	Cat# + company
Live Dead stain aqua	L34957, Thermo
Live/Dead stain FITC	L23101, Thermo
APC mouse anti-human IFN-g	554702, BD
Pacific Blue mouse anti-human CD8	558207, BD
CD8 Alexa488	557696, BD
CD25 PerCP Cy5.5	45-0259-42, ebio
CD69 Pe-Cy7	560712, BD
CD4 efluor 450	48-0048-42, ebio
CD3 PE	12-0037-42, ebio
Penta-His AF488	35310, Qiagen

Table S7. Tumor cell lines. Related to Star methods section "Cell lines"

Cell lines	Source / number
T2	ATCC/CRL-1992
MCF-7	ATCC/HTB-22
U266B1	ATCC/TIB-196
IM9	ATCC/CCL-159
Colo-205	ECACC 87061208
HCT116	ATCC CCL-247
NCI-H1755	ATCC/CRL-5892
NCI-H1703	ATCC/CRL-5889
MDA-MB231	ATCC/HTB-26

Supplemental References

1. Punjani, A., Rubinstein, J.L., Fleet, D.J., and Brubaker, M.A. (2017). cryoSPARC: algorithms for rapid unsupervised cryo-EM structure determination. Nat Methods *14*, 290–296. https://doi.org/10.1038/nmeth.4169.
2. Sanchez-Garcia, R., Gomez-Blanco, J., Cuervo, A., Carazo, J.M., Sorzano, C.O.S., and Vargas, J. (2021). DeepEMhancer: a deep learning solution for cryo-EM volume post-processing. Commun Biol *4*, 874. https://doi.org/10.1038/s42003-021-02399-1.
3. Terwilliger, T.C., Ludtke, S.J., Read, R.J., Adams, P.D., and Afonine, P. V (2020). Improvement of cryo-EM maps by density modification. Nat Methods *17*, 923–927. https://doi.org/10.1038/s41592-020-0914-9.
4. Terwilliger, T.C., Sobolev, O. V, Afonine, P. V, and Adams, P.D. (2018). Automated map sharpening by maximization of detail and connectivity. Acta Crystallogr D Struct Biol *74*, 545–559. https://doi.org/10.1107/S2059798318004655.
5. Afonine, P. V, Klaholz, B.P., Moriarty, N.W., Poon, B.K., Sobolev, O. V, Terwilliger, T.C., Adams, P.D., and Urzhumtsev, A. (2018). New tools for the analysis and validation of cryo-EM maps and atomic models. Acta Crystallogr D Struct Biol *74*, 814–840. https://doi.org/10.1107/S2059798318009324.
6. Krissinel, E., and Henrick, K. (2007). Inference of macromolecular assemblies from crystalline state. J Mol Biol *372*, 774–797. https://doi.org/10.1016/j.jmb.2007.05.022.
7. Dundas, J., Ouyang, Z., Tseng, J., Binkowski, A., Turpaz, Y., and Liang, J. (2006). CASTp: computed atlas of surface topography of proteins with structural and topographical mapping of functionally annotated residues. Nucleic Acids Res *34*, W116-8. https://doi.org/10.1093/nar/gkl282.
8. Case, D.A., Aktulga, H.M., Belfon, K., Cerutti, D.S., Cisneros, G.A., Cruzeiro, V.W.D., Forouzesh, N., Giese, T.J., Götz, A.W., Gohlke, H., et al. (2023). AmberTools. J Chem Inf Model *63*, 6183–6191. https://doi.org/10.1021/acs.jcim.3c01153.
9. Dyballa, N., and Metzger, S. (2009). Fast and sensitive colloidal coomassie G-250 staining for proteins in polyacrylamide gels. J Vis Exp. https://doi.org/10.3791/1431.
